# Synthesis and luminescence modulation of pyrazine-based gold(iii) pincer complexes†
†Electronic supplementary information (ESI) available: Details of synthesis and characterization, X-ray crystallography, photophysical properties, theoretical calculations. CCDC 1417819–1417822. For ESI and crystallographic data in CIF or other electronic format see DOI: 10.1039/c5cc07523h



**DOI:** 10.1039/c5cc07523h

**Published:** 2015-12-04

**Authors:** Julio Fernandez-Cestau, Benoît Bertrand, Maria Blaya, Garth A. Jones, Thomas J. Penfold, Manfred Bochmann

**Affiliations:** a School of Chemistry , University of East Anglia , Norwich , NR4 7TJ , UK . Email: m.bochmann@uea.ac.uk; b Department of Chemistry , Newcastle University , Newcastle upon Tyne , NE1 7RU , UK . Email: Tom.Penfold@newcastle.ac.uk

## Abstract

The first examples of pyrazine-based gold(iii) pincer complexes are reported; their intense photoemissions can be modified by protonation, *N*-alkylation or metal ions, without the need for altering the ligand framework. Emissions shift from red (77 K) to blue (298 K) due to thermally activated delayed fluorescence (TADF).

## Abstract 2

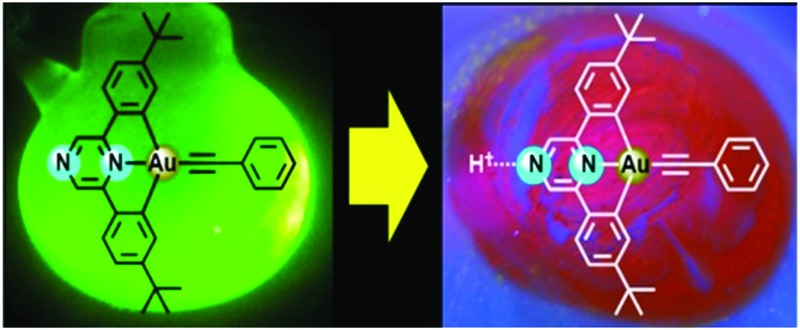
A second nitrogen for modulation: cyclometallated gold(iii) complexes based on pyrazine provide a new family of photoluminescent compounds which allow facile modulation of the emission wavelengths without the need for modifying the basic ligand framework.

Gold(iii) complexes with bis-cyclometallated ligands are characterized by their chemical stability and resistance to reduction. The ligand scaffold based on 2,6-diphenylpyridine^1^ has proved particularly useful in gold(iii) chemistry and forms (C^N^C)AuX pincer complexes of type **A** (Chart 1). Such complexes have proved to be highly versatile and, in combination with strong carbon-based σ-donor ligands (*e.g.* X = N-heterocyclic carbene, alkynyl), display interesting photophysical^2–4^ properties. This C^N^py^^C ligand system has also been successful in stabilizing types of compounds that have frequently been invoked as unstable intermediates in catalytic cycles or postulated in computer modelling of catalytic processes, such as gold(iii) hydrido, alkene, CO and peroxo complexes.^5^

**Chart 1:**
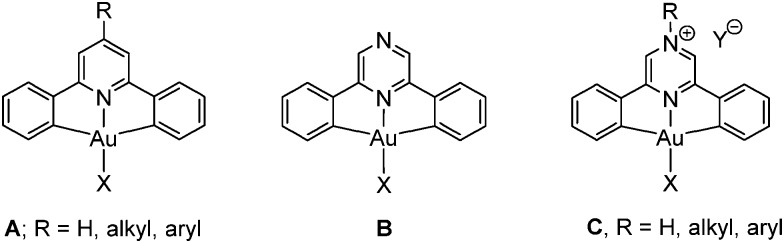


Photoemissive materials as components of electronic devices such as flat screen displays should ideally be capable of covering the whole range of the visible spectrum. Such changes in emission colours can be induced by suitable modification of the ligand framework. For (C^N^C)Au(iii) complexes a widely applied strategy for modulating the photoluminescence (PL) response and widening the range of emission wavelengths has been the introduction of electron donating or withdrawing substituents in the 4-position of the pyridine moiety.^3,4^ There are however limitations in this approach: firstly, the synthesis of C^N^C gold complexes involves two C–H activation steps, each of which is sensitive to the ligand structure and needs to be optimized for each new ligand; secondly, the modulation of electronic characteristics of the central pyridine moiety that can be achieved by inductive or mesomeric substituent effects is limited.

Rather more profound electronic changes in C^N^C ligands can be introduced by replacing the central pyridine ring by other heterocycles, such as pyrazine, to give compounds of type **B**. The lowest-energy π–π* transition in pyrazine is about 0.95 eV smaller than in pyridine;^6,7^ pyrazine ligands are therefore much better electron acceptors and are likely to produce a red-shift of their UV and photoemission wavelengths. In addition, pyrazine complexes **B** offer scope for further derivatisation by protonation or quaternisation of the non-coordinating N atom, to give salts of type **C**.

Complexes of aryl-substituted pyrazines and quinoxalines are of course well-known for iridium(iii) and platinum(ii), since for these metals they are readily accessible by direct cyclometallation of the neutral ligand precursors by noble metal halides.^8^ By contrast, related gold(iii) complexes have until now been inaccessible since the usual methods employed for the synthesis of pyridine complexes **A** fail for the analogous pyrazine derivatives. Here we report the first examples of cyclometallated Au(iii) pyrazine complexes and the facile modification of their photoluminescence properties.

The mercuration of the pro-ligand 2,6-bis(4′-*t*-BuC_6_H_4_)_2_pz (pz = pyrazine) requires forcing conditions but proceeds using Hg(tfa)_2_ in Htfa (tfa = CF_3_CO_2_) to give (C^N^pz^^C)HgCl·2Htfa (**1**). Transmetallation with KAuCl_4_ affords (C^N^pz^^C)AuCl (**2**) as a yellow crystalline powder in good yield (Scheme 1).

**Scheme 1:**
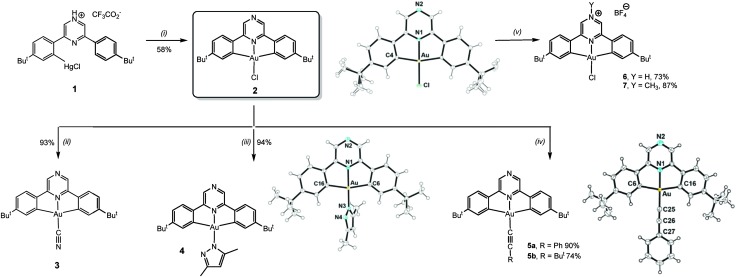
Conditions: (i) KAuCl_4_, CH_3_CN/H_2_O 1 : 1, reflux 72 h. (ii) KCN, CH_2_Cl_2_, 20 °C, 24 h. (iii) KO^*t*^Bu, 3,5-dimethylpyrazole, toluene, 20 °C, 6 h. (iv) AgC≡CR, CH_2_Cl_2_, 20 °C, 24 h. (v) HBF_4_·Et_2_O, Et_2_O, 20 °C, 30 min. (**6**), or [Me_3_O]BF_4_, toluene/1,2-C_6_H_4_F_2_ (5 : 1 v/v), 12 h. (**7**). Molecular structures [selected bond distances (Å) and angles (°)]: **2**: Au–N1 1.972(3), Au–Cl 2.2651(11), Au–C4 2.078; N1–Au–C4 81.28(8), C4–Au–Cl 98.72(8), C4–Au–C4′ 162.56(16), N1–Au–Cl 180. **4**: Au–N1 1.979(2), Au–C6 2.080(3), Au–C16 2.073(3), Au–N3 1.991(2); C6–Au–N1 81.39(10), C16–Au–N1 81.32(11), C6–Au–N3 98.88(10), C16–Au–N3 98.38(11), N1–Au–N3 178.33(9). **5a**: Au–N1 1.997(5), Au–C6 2.070(6), Au–C16 2.083(7), Au–C25 1.979(7), C25–C26 1.199(9); N1–Au–C6 80.4(2), N1–Au–C16 81.2(2), C6–Au–C25 99.3(3), C16–Au–C25 99.0(3), C6–Au–C16 161.6(3), Au–C25–C26 175.9(6), C25–C26–C27 177.4(7).

Substitution of the chloride ligand in **2** with KCN gives the cyanide complex **3**, while treatment with dimethylpyrazole affords the pyrazolato complex **4**. The reaction with AgC≡CR in CH_2_Cl_2_ affords the acetylides (C^N^pz^^C)AuC≡CR (**5a**, R = Ph; **5b**, R = Bu^*t*^) in essentially quantitative yield.

Pyrazine is only weakly basic (p*K*_a_ 1.30, *vs.* 5.20 of pyridine).^9^ However, protonation of **2** by HBF_4_·OEt_2_ generated the corresponding salt **6**. Remarkably, the protonation by dry HCl in Et_2_O proved to be reversible, and evaporation of the solvent from the HCl adduct regenerated neutral **2**. The alkylation of the non-coordinating pyrazine N-atom was achieved using Meerwein's salt [Me_3_O]BF_4_ to give **7** (Scheme 1) as a deep-red crystalline solid in 87% yield. The molecular structures^10^ of **2**, **4**, **5a** and **5b** were determined by X-ray diffraction (ESI†). The interatomic distances and angles are as expected for square-planar pincer complexes of this type (see ESI† for details).

All the complexes show moderate to intense luminescence in the solid state and in solution (for a detailed summary see the ESI,† Tables S2 and S3). Both the UV-vis spectra and the PL bands are characterized by the vibronic progression of the C^N^pz^^C pincer. As expected on the basis of the lower π–π* energy gap of the pyrazine compounds, the bands are red-shifted by about 40–50 nm compared with their C^N^py^^C analogues (see Fig. S22, ESI†).

Whereas in the case of diphenylpyridine-based complexes of type **A** strongly σ-donating ligands (such as aryls, acetylides or N-heterocyclic carbenes^3,4^) are required to raise the d-orbital energy of Au(iii) and increase the ^3^LLCT contribution to the emissive state, the pyrazine derivatives **2–7** reported here are emissive even if ligands X are weaker heteroatom donors. This is best exemplified by the chlorides: whereas (C^N^py^^C)AuCl is non-emissive at room temperature,^1^ complex **2** shows noticeable naked eye emission in fluid media (CH_2_Cl_2_ or 2-MeTHF) at 298 K that becomes very intense at 77 K (Fig. S20, ESI†). Similarly, the pyrazolato complex **4** is brightly emissive in the solid state or in fluid media at 298 K, while under identical conditions, the C^N^py^^C analogue is only weakly emissive.^11^ The pyrazine ligand framework of type **B** therefore significantly widens the range of photoemissive metal–ligand combinations.

In solution, the neutral complexes **2–5** all emit in the yellow to green region of the spectrum, mainly due to a triplet state based on the diphenylpyrazine pincer ligand. The excited state lifetimes show biexponential decay, with the fast component in the range of 5–20 ns.

At 77 K the emissions of **2**, both in the solid state (*λ*maxem 563 nm) and in solution (*λ*maxem = 532 nm), agree reasonably well with the value calculated for a T^1^ → S^0^ transition (*λ*calcem = 541 nm).‡
‡In the solid state at room temperature, complex **2** is non-emissive, most probably due to close intermolecular interactions in the crystal lattice which quench the luminescence. This notion is supported by the observed higher solid-state emission intensity of **5b** (*λ*_max_ = 523 nm, *φ* = 8.3) compared to **5a** (*λ*_max_ = 523 nm, *φ* = 4.5); the crystal packing shows that for **5b** intermolecular π-stacking is disfavoured on steric grounds. In solution at 298 K the PL intensities of **5a** (*λ*_max_ = 526 nm, *φ* = 0.462) and **5b** (*λ*_max_ = 526 nm, *φ* = 0.512) are essentially identical (see Fig. S19, ESI†). However, at 298 K the emission shows a significant blue-shift, to 482 nm, a feature that is even more pronounced on addition of acid (*vide infra*).

One of the most important challenges in the design of photoluminescent devices is the ability to modulate the energy of the emitted light. In the pyrazine ligand system, the easiest way to achieve this is by making use of the non-coordinating nitrogen of the pyrazine ring. Strong Brønsted and Lewis acids do indeed produce dramatic changes in PL response. In order to eliminate any possible anion effects, initial protonation studies were carried out using the solid Brønsted acid [H(OEt_2_)_2_][H_2_N{B(C_6_F_5_)_3_}_2_]^12^ (“HNB_2_”). As shown in Fig. 1a, protonating **2** with this acid results in a blue-shift compared with the neutral complex, from 482 to 458 nm. On the other hand, at 77 K the emissions of both the neutral and protonated compounds are remarkable similar (as expected since both the HOMO and LUMO are based on the pyrazine ligand and are equally affected by protonation) and the emission is due to an intra-ligand charge transfer (^3^ILCT) process.

**Fig. 1:**
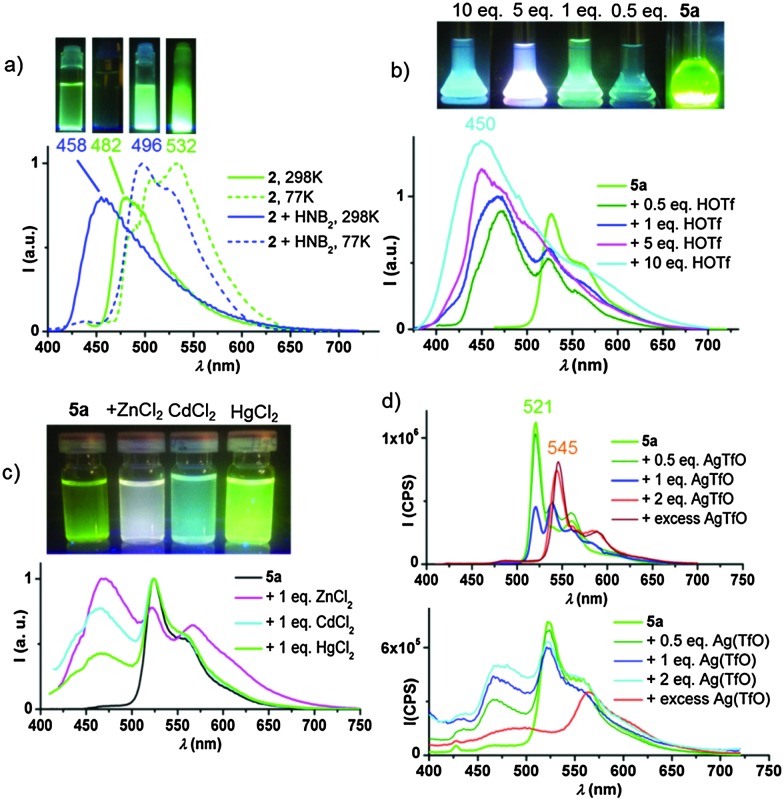
(a) PL of **2** (CH_2_Cl_2_, 1 × 10^–4^ M) in the absence and presence of two equivalents of [H(OEt_2_)_2_][H_2_N{B(C_6_F_5_)_3_}_2_] (HNB_2_) at 298 and 77 K. (b) PL response of **5a** (CH_2_Cl_2_, 1 × 10^–4^ M) to the addition of HOTf at 298 K. (c) PL of mixtures of **5a** with equimolar amounts of MCl_2_ (M = Zn, Cd, Hg) in THFMe-2 ([**5a**] = 10^–4^ M) at 298 K. (d) PL response of **5a** (THFMe-2, 5 × 10^–4^ M) to the addition of AgTfO at 77 (top) and 298 K (bottom).

The temperature-dependence of the photoluminescence response is in agreement with a TADF process,^13^ where the energy difference between the T^1^ and S^1^ excited states is sufficiently small to allow thermally driven repopulation of the S^1^ state from the T^1^ state, with the faster decay occurring *via* the S^1^ → S^0^ transition. In agreement with this mechanism, the quantum yields for the protonated pyrazine systems are about an order of magnitude higher than those of the neutral complexes. Photoemissions *via* a TADF mechanism are common for copper(i);^14^ but have not been previously reported for Au(iii).

The alkynyl complexes **5a** and **5b** show similar behaviour: the gradual addition of triflic acid results in the progressive growth of a blue emission (*λ*maxem = 450 nm for **5a**, Fig. 1b). Emissions involve a mixture of states, with both blue and green components, which combine to appear white.

The same effect can be achieved with the Lewis acid B(C_6_F_5_)_3_. In this case the high energy component appears at slightly lower energy (*λ*maxem = 481 nm), to give a more pronounced blue effect.

Whereas in the case of the chloride **2** both HOMO and LUMO were based on the pincer ligand, for **5a** the excitation involves metal-perturbed ligand-to-ligand π(C≡CPh) → π*(C^N^pz^^C) charge transfer (^3^LLCT, also involving HOMO–2). The effect of adding H^+^ or B(C_6_F_5_)_3_ to the pyrazine N-atom is therefore pronounced. DFT calculations predict a strong red shift on forming the **5a**-H^+^ cation since on protonation the HOMO–LUMO gap is reduced (*e.g.* calculated HOMO–LUMO difference for **5a** = 5.963 eV, compared to 4.878 eV after H^+^ addition). In agreement with this, solutions of **5a**·B(C_6_F_5_)_3_ at 77 K display an intense emission at 569 nm (Fig. S26, ESI†) which is gradually deactivated on warming, while at the same time a high energy component increases, resulting in the blue emission at *λ*maxem = 481 nm at 298 K. As can be seen (Fig. S29, ESI†), these reactions also occur in the solid state.

Different metal ions also lead to distinctive modulations of the PL emission, arising from a mixture of states. The addition of ZnCl_2_, CdCl_2_ and HgCl_2_ (1 : 1 molar ratio) results in white, turquoise and light green emissions, respectively (Fig. 1c). The gradual addition of ZnCl_2_ to a solution of the complex **5a** in 2-MeTHF, results in the activation of the blue TADF component at the expense of the green phosphorescence (see Fig. S27, ESI†). Cooling to 77 K recovers the phosphorescent emission.

The addition of AgOTf or CuOTf also enhances the emission *via* the TADF mechanism (see Fig. 1d for Ag^+^ and Fig. S28, ESI† for Cu^+^). Freezing 2-MeTHF solutions of **5a**/AgOTf to 77 K reveals the presence of two different systems; complex **5a** (*λ*maxem = 521 nm), and a second complex characterized by a band with maximum at 545 nm. The mixture of **5a** with two equivalents and with an excess of Ag^+^ both show the same emission band, while 1 equivalent of Ag^+^ gives two bands of about equal intensity. For these reasons, we tentatively suggest the formation of an aggregate of **5a** with two Ag^+^ ions (*e.g.* coordination of two Ag^+^ ions to the C≡C bond and the pyrazine-N atom may be envisaged).

The *N*-methylated complex **7** mirrors the behaviour of the H^+^ adducts and shows blue emission in solution (*λ*maxem = 460 nm) but is dark-red in the solid state (*λ*_em_ = 623_max_, 680sh).

In summary, cyclometallated gold(iii) pincer complexes based on pyrazine provide a new family of photoluminescent compounds which allow facile modulation of the emission characteristics by protonation, alkylation, Lewis acids or metal ions, without the need for modifying the pincer ligand framework. The modulation arises from the coexistence of high energy TADF and ^3^IL (C^N^pz^^C)/^3^LLCT (X → C^N^pz^^C) transitions.

This work was supported by the European Research Council. M. B. is an ERC Advanced Investigator Award holder (grant no. 338944-GOCAT).
