# Supplementary material for: Synthesis and luminescence modulation of pyrazine-based gold(iii) pincer complexes
Source: Chem Commun (Camb). 2015 Dec 4;51(93):16629–32. doi: 10.1039/c5cc07523h (PMC4672750; doi:10.1039/c5cc07523h)
Supplement: Supplementary file 1 [file CC-051-C5CC07523H-s001.pdf]

Electronic Supplementary Material (ESI) for Chemical Communications.
This journal is © The Royal Society of Chemistry 2015

Synthesis and Luminescence Modulation of Pyrazine-Based Gold(III) Pincer Complexes

Julio Fernandez-Cestau,[a] Benoît Bertrand,[a] Maria Blaya,[a] Garth A. Jones,[a] Thomas J. Penfold[b]* and Manfred Bochmann[a]*

[a] *School of Chemistry, University of East Anglia, Norwich, NR4 7TJ, UK. Tel: +44 016035 92044; E-mail: m.bochmann@uea.ac.uk*
[b] *Department of Chemistry, Newcastle University, Newcastle upon Tyne, NE1 7RU, UK.*

Supporting Information

Synthesis and characterization **S1**

X-ray crystallography **S2**

Photophysical properties **S3**

Theoretical calculations **S4**

References **S5**

S1. Synthesis and Characterization

When required, manipulations were performed using standard Schlenk techniques under dry nitrogen or an M. Braun glove box. Nitrogen was purified by passing through columns of supported P_2O_5 with moisture indicator, and of activated 4 Å molecular sieves. Anhydrous solvents were freshly distilled from appropriate drying agents. IR spectra were recorded using a Perkin Elmer Spectrum 65 FT-IR spectrometer with a diamond ATR attachment. Elemental analyses were carried out at London Metropolitan University.

^{1}H, ^{13}C{^{1}H} and ^{19}F spectra were recorded using a Bruker Avance DPX-300 spectrometer. $CDCl_3$, CD_2Cl_2 and DMSO-d^6 were dried over CaH_2, degassed by three freeze-pump-thaw cycles and stored on 4 Å molecular sieves prior to use. ^{1}H NMR spectra (300.13 MHz) were referenced to the residual protons of the deuterated solvent used. ^{13}C{^{1}H} NMR spectra (75.47 MHz) were referenced internally to the D-coupled ^{13}C resonances of the NMR solvent. ^{19}F{^{1}H} NMR spectra (282.38 MHz) were referenced to $CFCl_3$.

Photoluminescence measurements were recorded on a Perkin Elmer LS55 Fluorescence Spectrometer with a solids mount attachment where appropriate. Time resolved fluorescence data were collected on a TCSPC Fluorolog Horiba Jobin Yvon spectrofluorimeter using Horiba Jobin Yvon DataStation v2.4 software. A NanoLED of 370 nm was used as excitation source. The collected data were analysed using a Horiba Jobin Yvon DAS6 v6.3 software.

Synthesis of 2,6-bis(4’-tertbutylphenyl)pyrazine

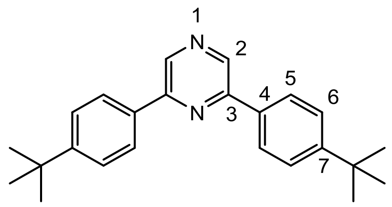

The synthesis of the ligand was carried out by slight modification of a literature method.[S1] 2,6-Dichloropyrazine (2.00 g, 13.4 mmol), 4-*t*-butylphenylboronic acid (4.78 g, 26.8 mmol), Na_2CO_3 (2.85 g, 26.8 mmol) and $Pd(PPh_3)_2Cl_2$ (0.056 g, 0.08 mmol, 0.6%) were added to a round-bottomed flask. MeCN (40 mL) and H_2O (40 mL) were added and N_2 bubbled through the mixture for 15 min, followed by refluxing under N_2 for 48 h. The volume of the mixture was reduced to ca 30 mL. A white solid precipitated which was filtered off, washed with H_2O (3 × 20 mL) and EtOH (3 × 5 mL) and air dried, yielding the title compound in good yield (3.96 g, 11.5 mmol, 86 % yield). Anal. Calcd for $C_{24}H_{28}N_2$ (344.5): C, 83.68; H, 8.19; N, 8.13. Found: C, 83.45; H, 8.35; N, 8.15. ^{1}H NMR (300 MHz, $CDCl_3$, 293 K): δ 8.92 (s, 2H, H^2), 8.09 (d, $^{3}J_{H-H}$ = 8.4 Hz, 4H, H^5), 7.56 (d, $^{3}J_{H-H}$ = 8.4 Hz, 4H, H^6), 1.39 (s, 18H, $-CH_3$, ^{t}Bu). ^{13}C{^{1}H} NMR (75 MHz, $CDCl_3$, 293 K) δ

153.2 (^{4}C), 151.6 (^{4}C), 139.4 (CH), 133.9 (^{4}C), 126.8 (CH), 126.0 (CH), 34.8 (*C*Me$_3$), 31.3 (C*Me*$_3$).

Synthesis of (C^N^{pz}^C)HgCl·2HOAc^F (1)

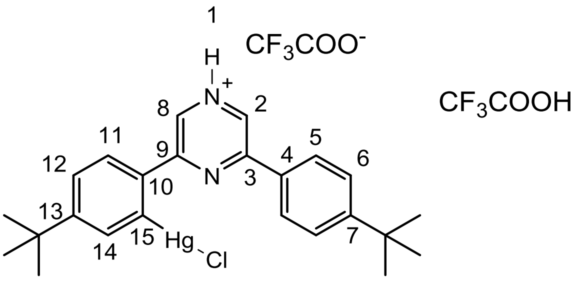

A mixture of 2,6-bis(4'-*tert*.-butylphenyl)pyrazine (3.00 g, 8.7 mmol) and Hg(OAc^F)$_2$ (4.40 g, 8.7 mmol) were dissolved into trifluoroacetic acid (20mL) and refluxed for 48 h. LiCl (0.38 g, 8.7 mmol) dissolved in hot methanol was added, leading to the formation of a white precipitate. The mixture was then stirred at room temperature for 1 h. The precipitate was filtered and washed with water and methanol. After drying under vacuum the pure product was obtained as a beige powder (4.50 g, 5.6 mmol, 64 % yield). Anal. Calcd for $C_{24}H_{27}N_2HgCl \cdot 2HOAc^F$ (808.1): C, 41.61; H, 3.62; N, 3.47. Found: C, 41.30; H, 4.28; N, 4.26. ^{1}H NMR (DMSO-*d*^{6}, 300.13 MHz): δ 9.22 (s, 1 H, H$^{2/8}$), 9.18 (broad s, 1 H, NH$^+$), 9.13 (s, 1 H, H$^{2/8}$), 8.18 (d, $^3J_{H\text{-}H}$ = 8.2 Hz, 2 H, H^5), 8.01 (d, $^3J_{H\text{-}H}$ = 8.2 Hz, 1 H, H^{11}), 7.85 (dd, $^3J_{H\text{-}Hg}$ = 3.8 Hz, $^4J_{H\text{-}H}$ = 0.6 Hz, 1 H, H^{14}), 7.54 (d, $^3J_{H\text{-}H}$ = 8.2 Hz, 2 H, H^6), 7.45 (m, 1 H, H^{12}), 1.34 (s, 18 H, ^{t}Bu). ^{13}C{^{1}H} NMR (DMSO-*d*^{6}, 75.48 MHz): δ 152.9, 152.7, 152.6, 152.0, 151.8, 150.3, 141.8, 141.4, 140.4, 137.5, 137.2, 135.0, 134.6, 132.5, 128.1, 128.0, 127.2, 125.6, 125.1, 124.9, 79.3, 78.9, 34.6, 34.4, 31.0, 30.9. ^{19}F{^{1}H} NMR (DMSO-*d*^{6}, 282.38): δ -73.4 (s, CF_3COO^-).

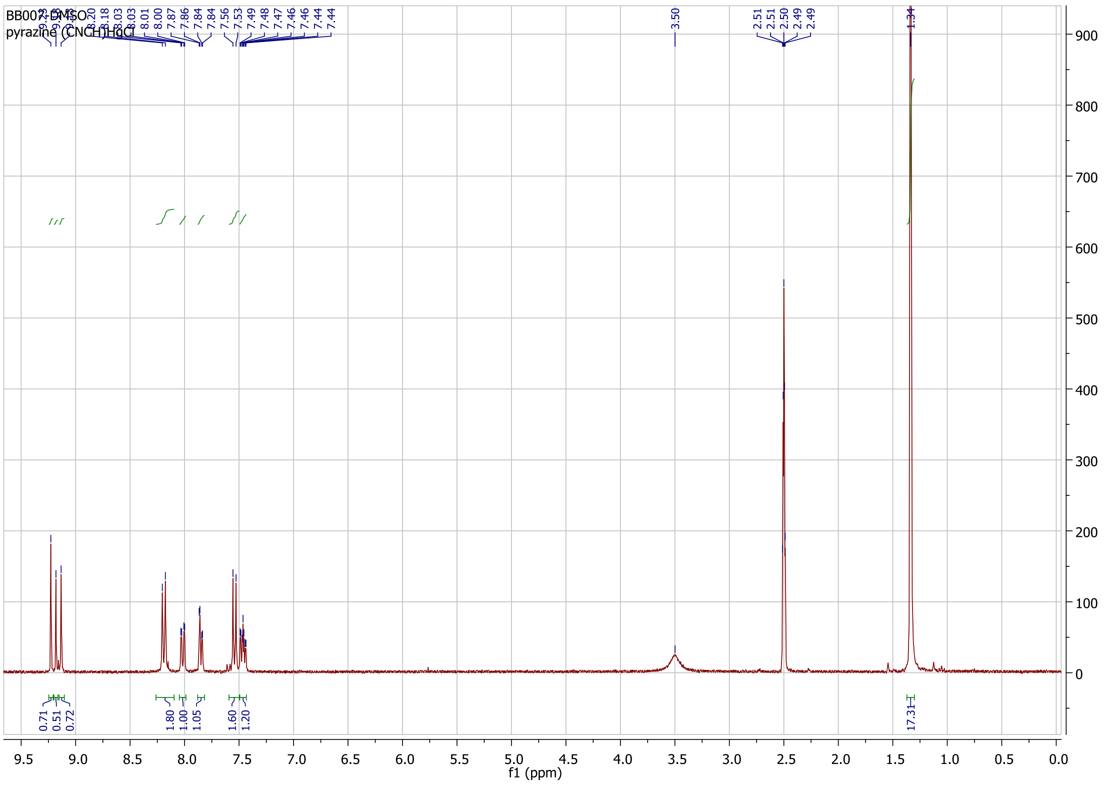

Figure S1. ^{1}H NMR spectrum of **1**.

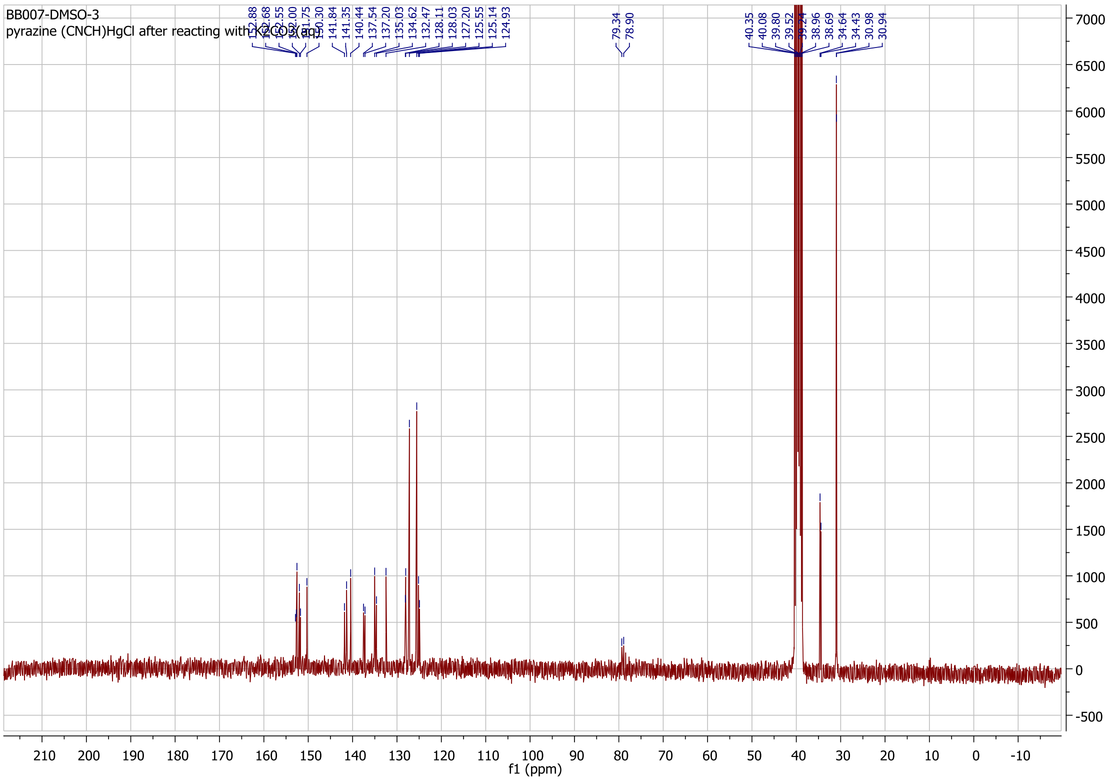

Figure S2. ^{13}C NMR spectrum of **1**.

Synthesis of (C^N^{pz}^C)AuCl (2)

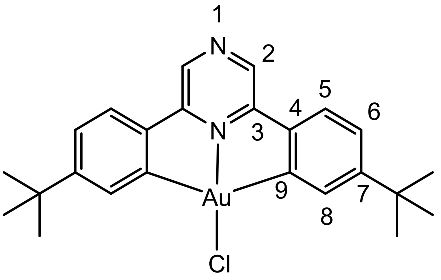

(C^N^{pz}^CH)HgCl·2HOAc^{F} (**1**) (0.79 g, 1.14 mmol) and K[$AuCl_4$] (0.43 g, 1.14 mmol) were stirred in a mixture MeCN/water: 1/1 (30 mL) at reflux for 72 h. After the mixture had cooled to room temperature, the crude product, a pale orange precipitate, was filtered off and washed with water (2 × 20 mL) and MeCN (20 mL) of and dried under vacuum. For further purification this orange powder was dissolved in DMSO (50 mL) and stirred at 120 °C for 2 h. The solution was cooled to room temperature and water (100 mL) was added, causing the precipitation of a yellow product. The mixture was filtered through celite. The residue was washed with water, followed by acetone and then dichloromethane. A yellow solution was obtained. The solvents were evaporated. The yellow residue was dissolved in CH_2Cl_2 (200 mL) and washed with water (3 × 50 mL). The organic phase was dried with Na_2SO_4 and the solvent evaporated. The product was washed with cold hexane to afford pure **2** as a yellow powder (0.38 g, 0.66 mmol, 58 % yield). Anal. Calcd for $C_{24}H_{26}N_2AuCl$ (574.9): C, 50.14; H, 4.56; N, 4.87. Found: C, 50.15; H, 4.49; N, 4.97. ^{1}H NMR (CD_2Cl_2, 300.13 MHz): δ 8.75 (s, 2 H, H^2), 7.91 (d, $^4J_{H\text{-}H}$ = 1.9 Hz, 2 H, H^8), 7.60 (d, $^3J_{H\text{-}H}$ = 8.2 Hz, 2 H, H^5), 7.35 (dd, $^3J_{H\text{-}H}$ = 8.2 Hz, $^4J_{H\text{-}H}$ = 1.9 Hz, 2 H, H^6), 1.37 (s, 18 H, ^{t}Bu). ^{13}C{^{1}H} NMR (75 MHz, CD_2Cl_2, 293 K): δ 172.0 (s, C^9), 158.0 (s, $C^{3/4}$), 157.5 (s, $C^{3/4}$), 143.5 (s, C^7), 139.0 (s, C^2), 131.5 (s, C^8), 126.0 (s, C^5), 125.2 (s, C^6), 36.2 (s, *C*Me_3), 31.4 (s, C*Me*$_3$).

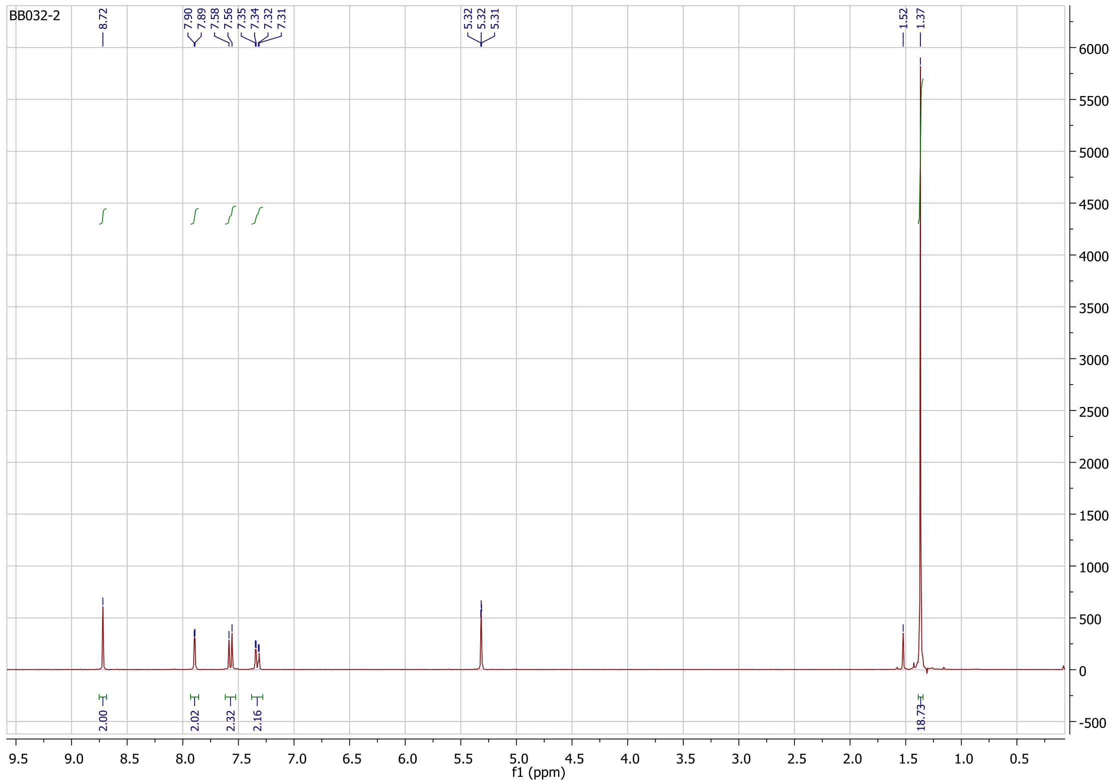

Figure S3. ^{1}H NMR spectrum of **2**.

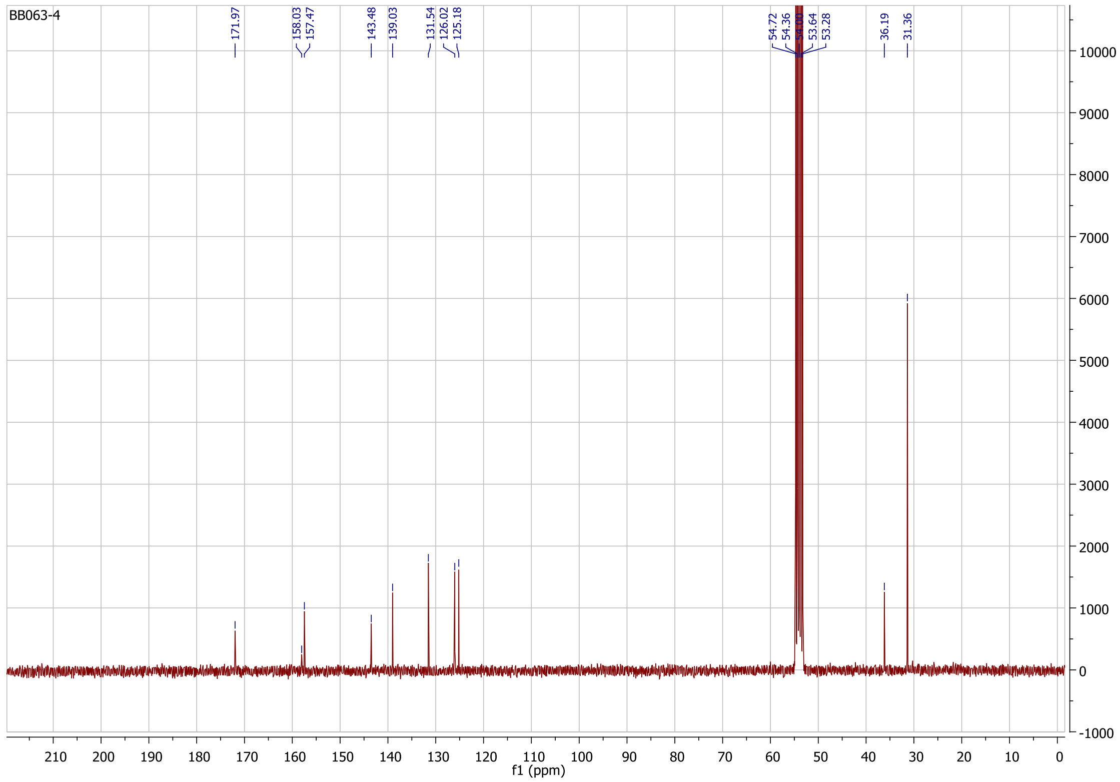

Figure S4. ^{13}C NMR spectrum of **2**.

Synthesis of (C^N^{pz}^C)AuC≡N (3)

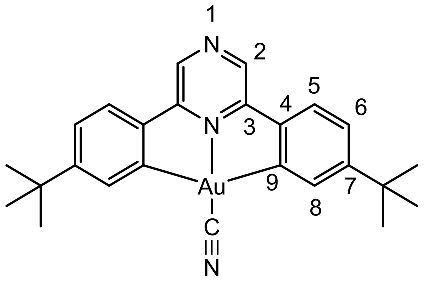

To a solution of (C^N^{pz}^C)AuCl **2** (0.06 g, 0.10 mmol) in CH_2Cl_2 (10 mL) was added NaCN (8 mg, 0.16 mmol). The mixture was stirred vigorously for 24 h and filtered through celite. The filtrate was evaporated to dryness and the residue treated with light petroleum to afford the product as a deep-yellow solid (0.055 g, 0.093 mmol, 93% yield). Anal. Calcd for $C_{25}H_{26}N_3Au$ (565.5): C, 53.10; H, 4.63; N, 7.43. Found: C, 53.40; H, 4.82; N, 7.22. IR (cm^{-1}): ν(C≡N) 2174 (sh), 2165 (w). ^{1}H NMR (300 MHz, CD_2Cl_2, 293 K): δ 8.74 (s, 2 H, H^2), 7.90 (d, $^4J_{H-H}$ = 2.0 Hz, 2 H, H^8), 7.59 (d, $^3J_{H-H}$ = 8.2 Hz, 2 H, H^5), 7.34 (dd, $^3J_{H-H}$ = 8.2, $^4J_{H-H}$ = 2.0 Hz, 2 H, H^6), 1.36 (s, 18 H, ^{t}Bu). ^{13}C{^{1}H} NMR (75 MHz, $CDCl_3$, 293 K) δ 167.6 (s, C^9), 158.5 (s, $C^{3/4}$), 158.3 (s, $C^{3/4}$), 144.2 (s, C^7), 139.2 (s, C^2), 135.2 (s, C^8), 126.4 (s, C^5), 125.3 (s, C^6), 36.1 (s, *C*Me$_3$), 31.3 (s, C*Me*$_3$), *C*N not visible.

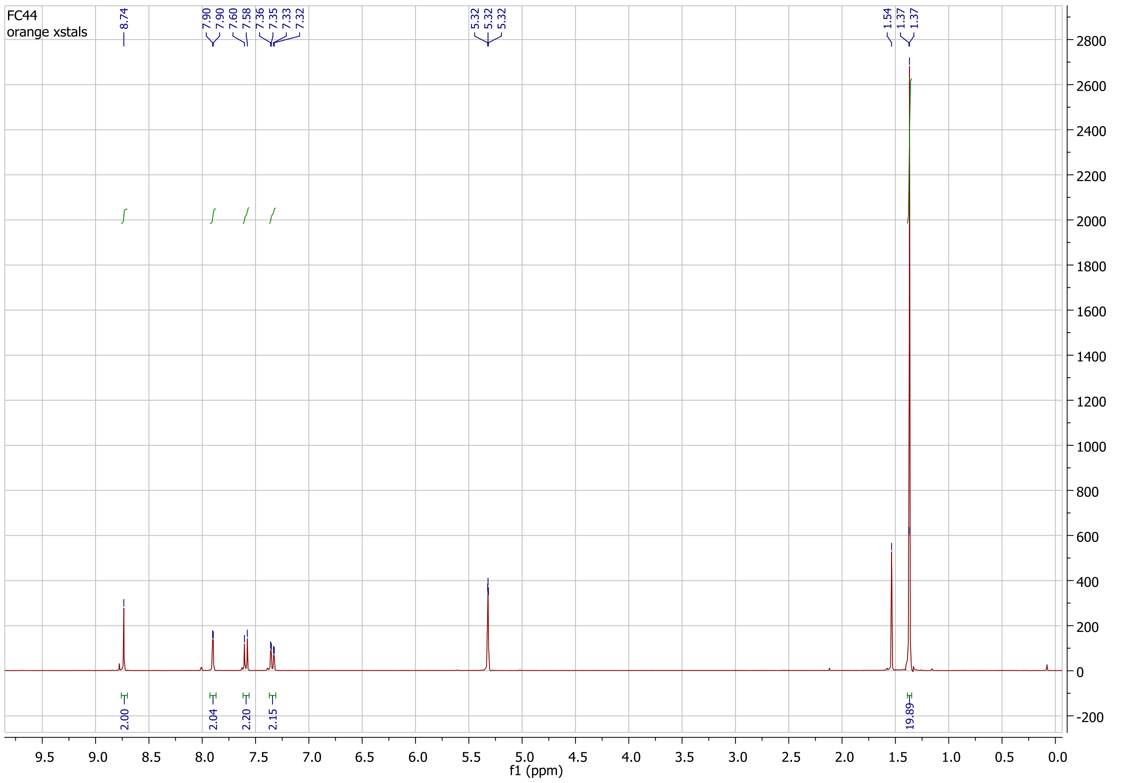

Figure S5. ^{1}H NMR spectrum of **3**.

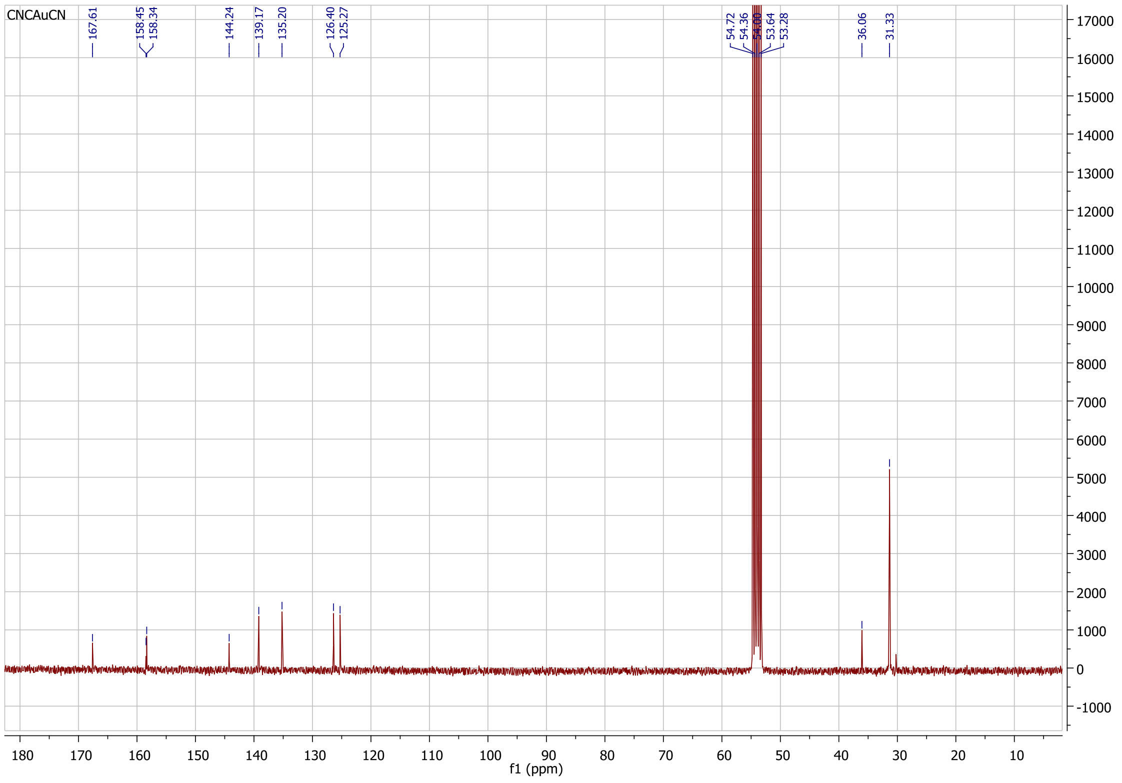

Figure S6. ^{13}C NMR spectrum of **3**.

Synthesis of (C^N^{pz}^C)Au(3,5-dimethylpyrazolyl) (4)

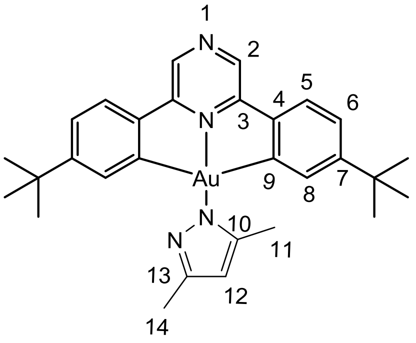

A mixture of (C^N^{pz}^C)AuCl **2** (0.050 g, 0.087 mmol), KO^{t}Bu (0.012 g, 0.104 mmol) in toluene (5 mL) was stirred for 3 h under an N_2 atmosphere at room temperature. 3,5-Dimethylpyrazole (0.009 g, 0.087 mmol) was added stirring was continued for another 3 h. The solution was filtered through celite. Removal of the solvent *in vacuo* gave the pure product as a yellow powder (0.052 g, 0.082 mmol, 94 % yield). Anal. Calcd for $C_{29}H_{33}N_4Au$ (634.2): C, 54.89; H, 5.24; N, 8.83. Found: C, 54.84; H, 5.31; N, 8.74. ^{1}H NMR (CD_2Cl_2, 300.13 MHz): 8.78 (s, 2 H, H^2), 7.63 (d, $^3J_{H\text{-}H}$ = 8.2 Hz, 2 H, H^5), 7.32 (dd, $^3J_{H\text{-}H}$ = 8.2 Hz, $^4J_{H\text{-}H}$ = 1.9 Hz, 2 H, H^6), 7.27 (d, $^4J_{H\text{-}H}$ = 1.9 Hz, 2 H, H^8), 6.02 (s, 1 H, H^{12}), 2.34 (s, 3 H, H$^{11/14}$), 2.27 (s, 3 H, H$^{11/14}$), 1.27 (s, 18 H, ^{t}Bu). ^{13}C{^{1}H} NMR (CD_2Cl_2, 75.48 MHz): 170.8 (s, C^9), 158.3 (s, $C^{3/4}$), 157.1 (s, $C^{3/4}$), 150.2 (s, C^7), 143.6 (s, $C^{10/13}$), 143.2 (s, $C^{10/13}$), 138.7 (s, C^2), 132.7 (s, C^8), 125.7 (s, C^5), 124.9 (s, C^6), 103.9 (s, C^{12}), 35.8 (s, *C*Me$_3$), 31.1 (s, C*Me*$_3$), 14.5 (s, $C^{11/14}$), 14.1 (s, $C^{11/14}$).

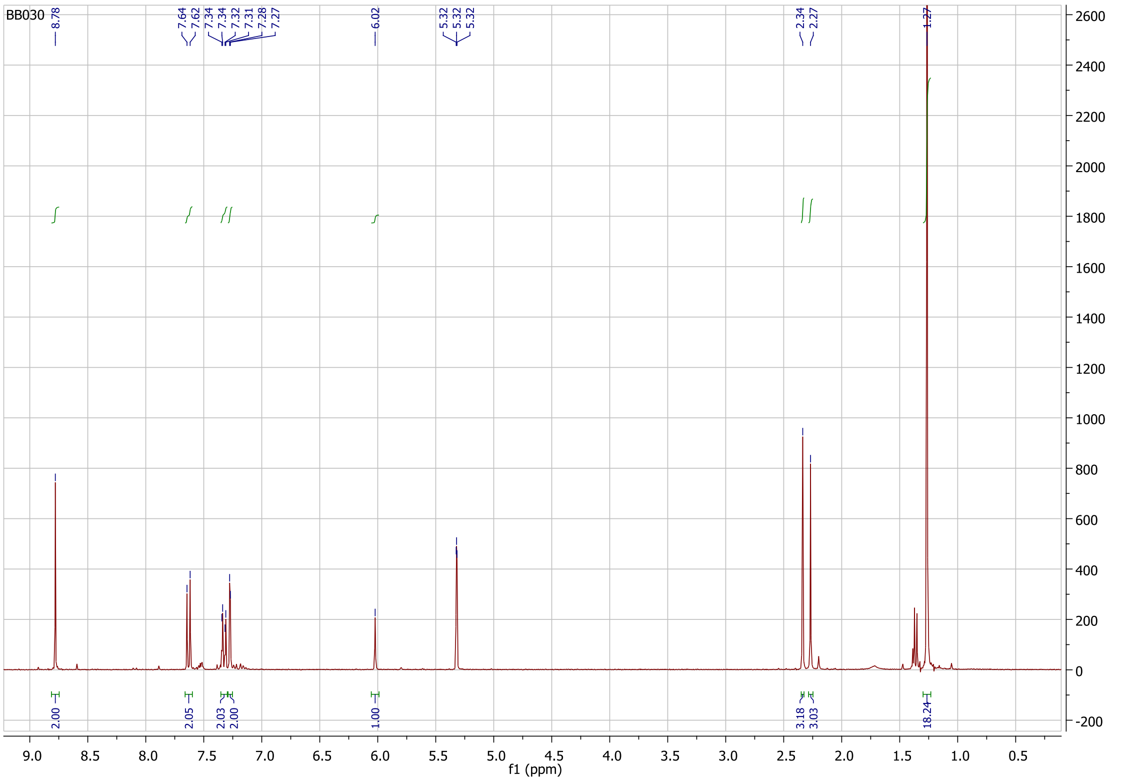

Figure S7. ^{1}H NMR spectrum of **4**.

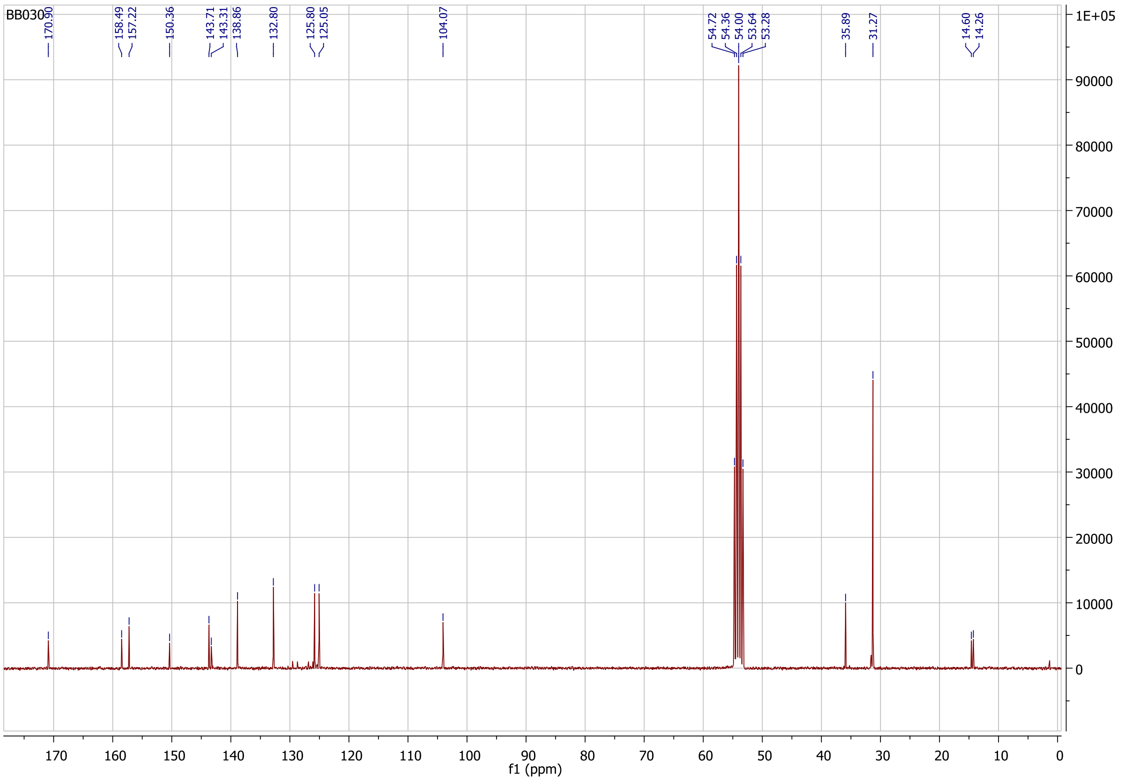

Figure S8. ^{13}C NMR spectrum of **4**.

Synthesis of (C^N^{pz}^C)AuC≡CPh (5a)

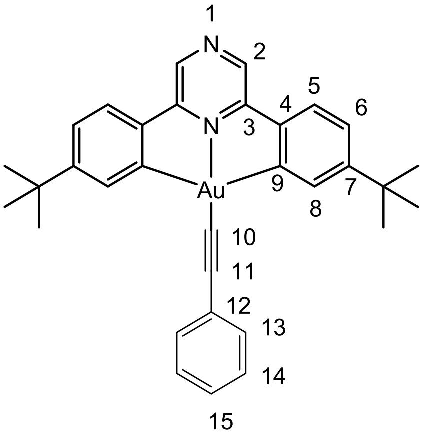

(C^N^{pz}^C)AuCl **2** (0.060 g, 0.10 mmol) and AgC≡CPh (0.033 g, 0.16 mmol) were charged into a vial and protected from the light. Dichloromethane (10 mL) was added. The mixture was sonicated for 1 h and stirred vigorously for 24 h before being filtered through celite. The filtrate was evaporated to dryness. The residue was treated with petroleum to give the product as a yellow solid (0.058 g, 0.090 mmol, 90% yield). Anal. Calcd for $C_{32}H_{31}N_2Au$ (640.6): C, 60.00; H, 4.88; N, 4.37. Found: C, 59.98; H, 4.90; N, 4.48. IR (cm^{-1}): ν(C≡C) 2149 (w). ^{1}H NMR (300 MHz, CD_2Cl_2): δ 8.83 (s, 2 H, H^2), 8.16 (d, $^4J_{H-H}$ = 2.0 Hz, 2 H, H^8), 7.65 (d, $^3J_{H-H}$ = 8.2 Hz, 2 H, H^5), 7.59 (dd, $^3J_{H-H}$ = 8.1, $^4J_{H-H}$ = 1.5 Hz, H^{13}), 7.40–7.31 (m, 5 H, H^6 + H^{14} + H^{15}), 1.39 (s, 18 H, ^{t}Bu). ^{13}C{^{1}H} NMR (75 MHz, $CDCl_3$, 293 K): δ 168.3 (s, C^9), 157.9 (s, $C^{3/4}$), 157.0 (s, $C^{3/4}$), 144.8 (s, C^7), 138.9 (s, C^2), 134.3 (s, C^8), 132.4 (s, C^{13}), 128.8 (s, C^{14}), 127.7 (s, C^{15}), 126.9 (s, C^{12}), 126.0 (s, C^5), 124.7 (s, C^6), 102.1 (s, C^{10}), 91.7 (s, C^{11}), 36.0 (s, $C(CH_3)_3$), 31.4 (s, CMe_3).

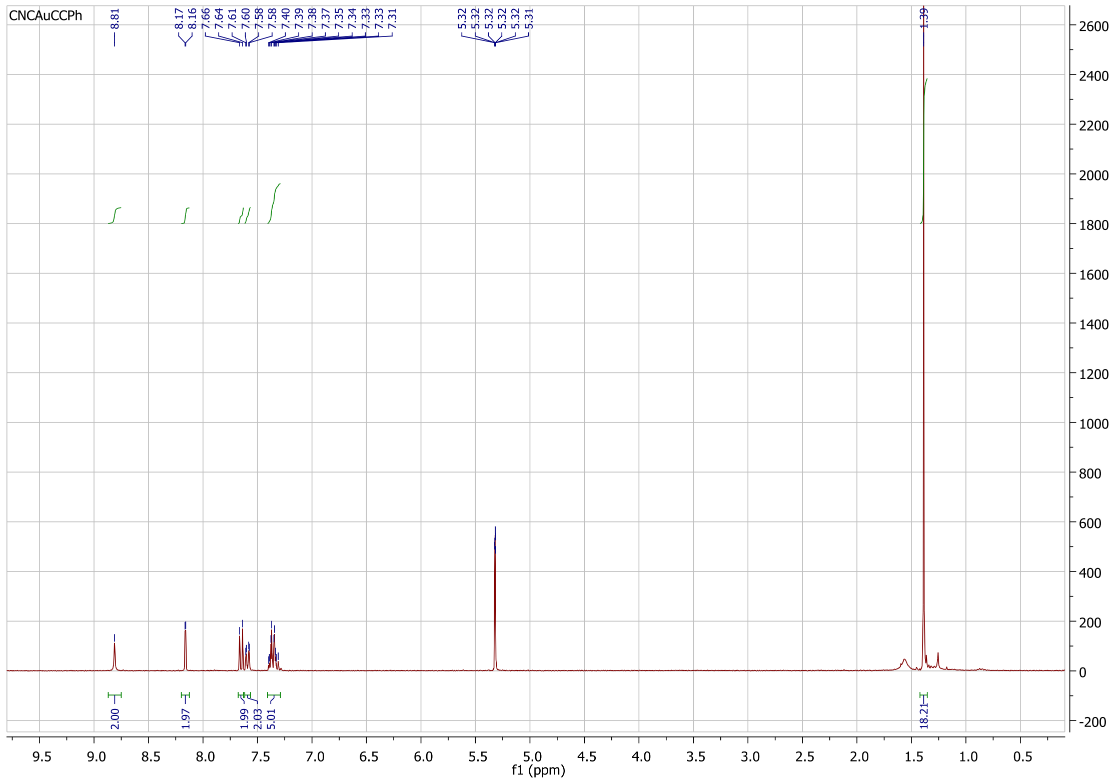

Figure S9. ^{1}H NMR spectrum of **5a**.

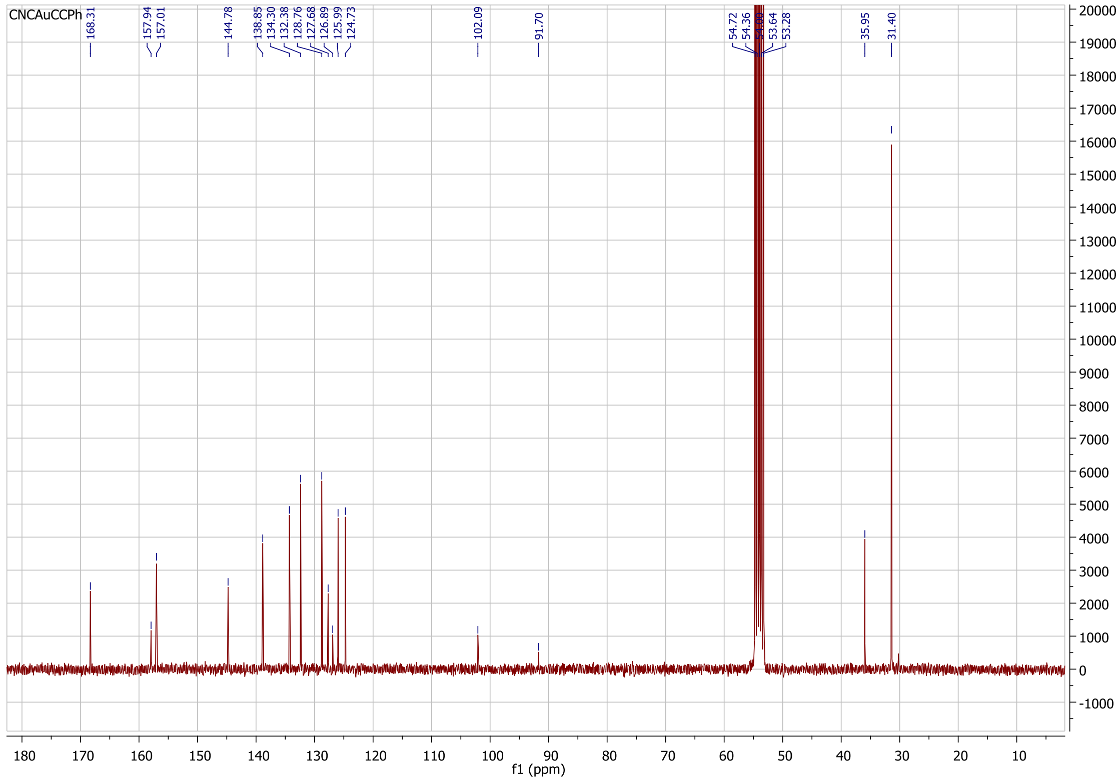

Figure S10. ^{13}C NMR spectrum of **5a**.

Synthesis of (C^N^{pz}^C)AuC≡C^{t}Bu (5b)

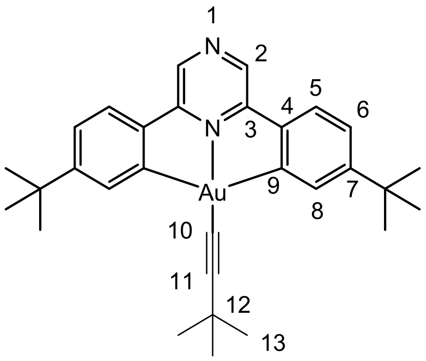

Following the procedure for **6a**, the complex was made from (C^N^{pz}^C)AuCl **2** (0.060 g, 0.10 mmol) and AgC≡C^{t}Bu (0.030 g, 0.16 mmol) as a yellow solid (0.045 g, 73% yield). Anal. Calcd for $C_{30}H_{35}N_2Au$ (620.6): C, 58.06; H, 5.68; N, 4.51. Found: C, 58.48; H, 4.90; N, 4.48. IR (cm^{-1}): ν(C≡C) 2090 (br). ^{1}H NMR (300 MHz, CD_2Cl_2): δ 8.78 (s, 2 H, H^2), 8.05 (d, $^4J_{H-H}$ = 2.0 Hz, 2 H, H^8), 7.62 (d, $^3J_{H-H}$ = 8.2 Hz, 2 H, H^5), 7.32 (dd, $^3J_{H-H}$ = 8.1, $^4J_{H-H}$ = 1.5 Hz, 2 H, H^6), 1.41 (s, 9 H, H^{13}), 1.37 (s, 18 H, ^{t}Bu). ^{13}C{^{1}H} NMR (75 MHz, $CDCl_3$, 293 K) δ 168.4 (s, *C*^{9}), 157.9 (s, *C*$^{3/4}$), 156.7 (s, *C*$^{3/4}$), 144.8 (s, *C*^{7}), 138.7 (s, *C*^{2}), 134.2 (s, *C*^{8}), 125.8 (s, *C*^{5}), 124.5 (s, *C*^{6}), 110.9 (s, *C*^{10}), 77.2 (s, *C*^{11}), 36.0 (s, *C*$(CH_3)_3$), 32.4 (s, *C*^{13}), 31.4 (s, C*Me*$_3$), 29.7 (s, *C*^{12}).

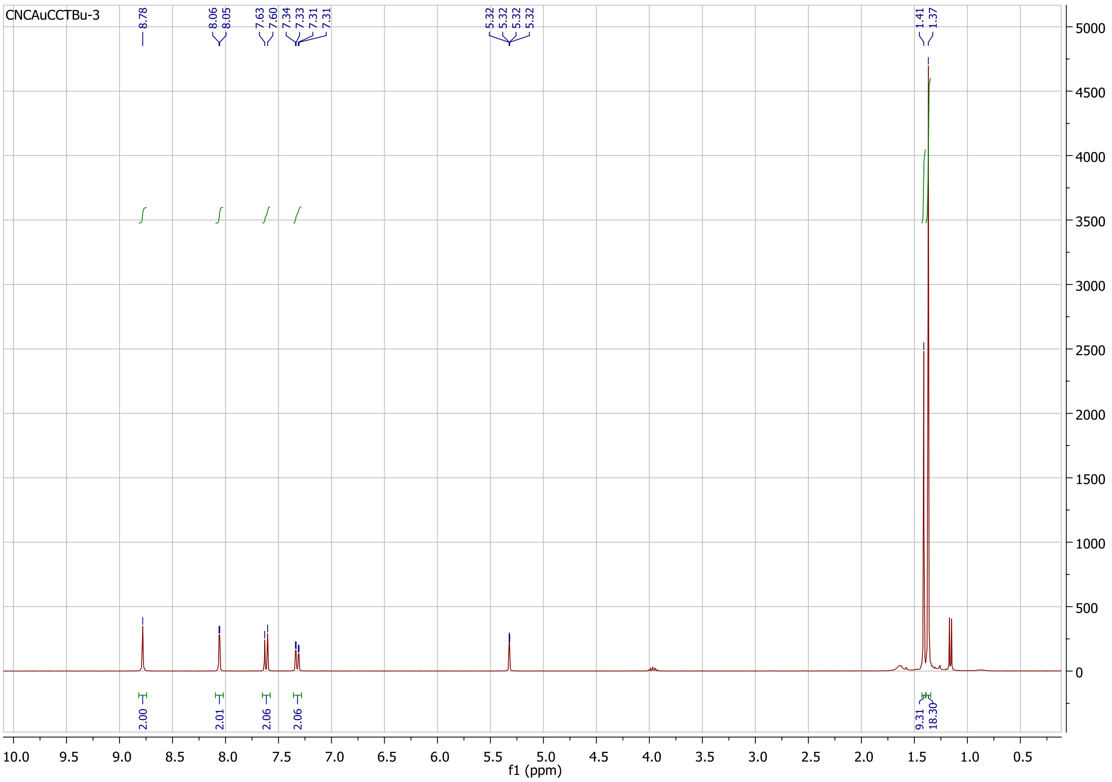

Figure S11. ^{1}H NMR spectrum of **5b**.

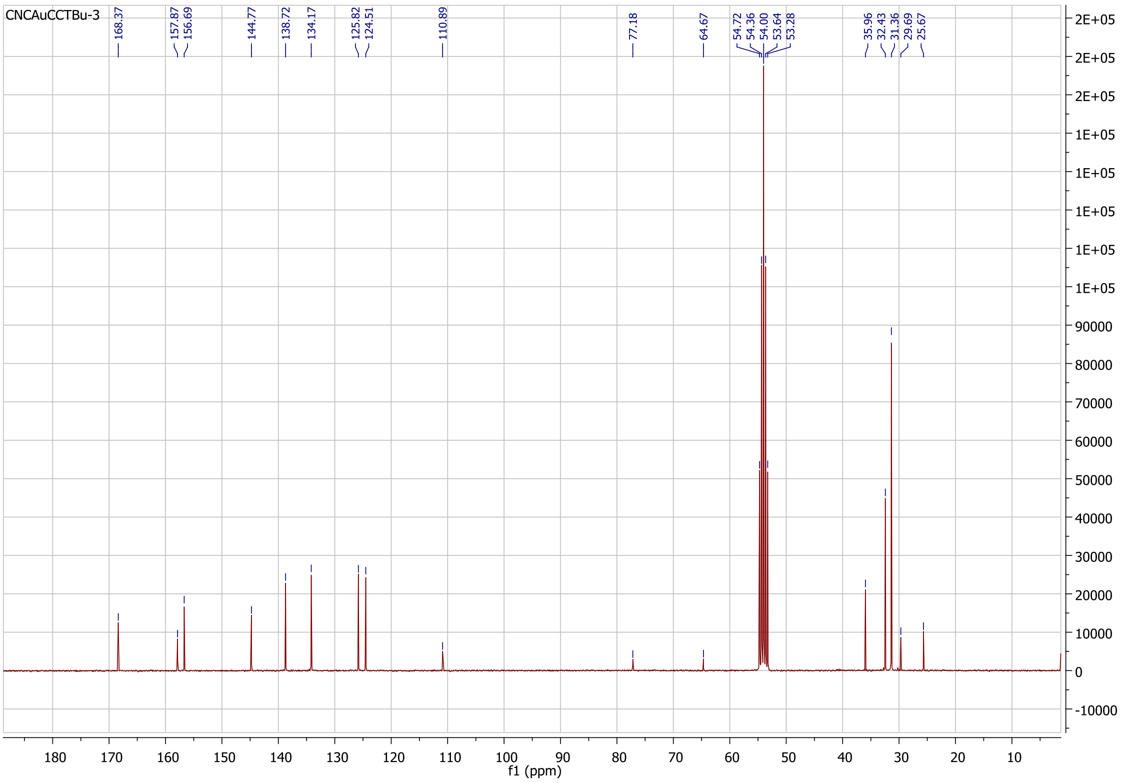

Figure S12. ^{13}C NMR spectrum of **6b**.

Synthesis of [(N-HC^N^{pz}^C)AuCl]BF$_4$ (6)

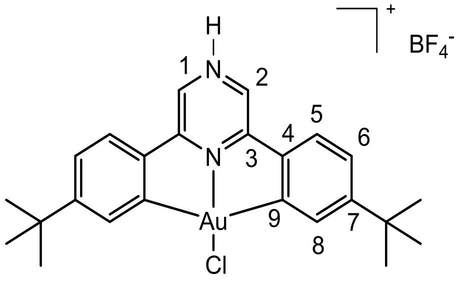

(C^N^{pz}^C)AuCl **2** (0.050 g, 0.087 mmol) was stirred at room temperature under N_2 atmosphere in distilled diethyl ether (5 mL). To this was added $HBF_4 \cdot Et_2O$ (0.012 mL, 0.087 mmol), leading to an immediate colour change from yellow to orange. The mixture was stirred for 30 min at room temperature. After evaporation of the solvent the red solid residue was suspended in dichloromethane. The addition of hexane led to the precipitation of a red product which was filtrated, washed with hexane (3 × 5 mL) and dried to afford the pure product as a red powder (0.042 g, 0.064 mmol, 73 % yield).Anal. Calcd for $C_{24}H_{27}N_2AuClBF_4$ (662.2): C, 43.50; H, 4.11; N, 4.23. Found: C, 43.63; H, 4.18; N, 4.37. The compound is only soluble in DMSO. In this solvent the spectroscopic data are similar to those of **2**.

Synthesis of [(N-MeC^N^{pz}^C)AuCl]BF$_4$ (7)

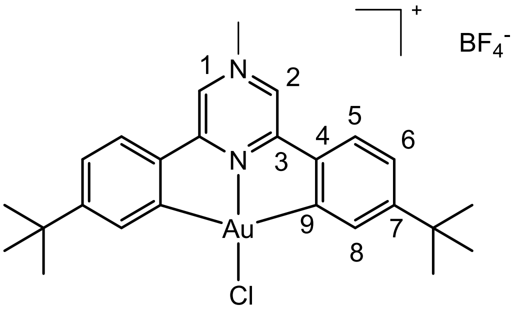

Under an N_2 atmosphere, a mixture of (C^N^{pz}^C)AuCl **2** (0.050 g, 0.087 mmol), $[Me_3O]BF_4$ (0.026 mg, 0.174 mmol), toluene (5 mL) and 1,2-difluorobenzene (1 mL) was stirred at room temperature overnight, leading to the formation of a deep-red precipitate. The solution was filtered through celite and the red solid component washed with cold toluene. The red product was recovered by washing the celite with acetone. After partial removal of the acetone and addition of diethylether, a deep red precipitate which was washed water (4 × 5 mL) and dried to afford the pure product as a red powder (0.051 g, 0.076 mmol, 87 % yield). Anal. Calcd for $C_{25}H_{29}N_2AuClBF_4$ (676.7): C, 44.37; H, 4.32; N, 4.14. Found: C, 44.23; H, 4.17; N, 4.08. ^{1}H NMR (acetone-d^6, 300.13 MHz): δ 9.62 (broad s, 2 H, H^2), 7.98 (d, $^3J_{H\text{-}H}$ = 8.4 Hz, 2 H, H^5), 7.94 (d, $^4J_{H\text{-}H}$ = 1.8 Hz, 2 H, H^8), 7.56 (d, $^3J_{H\text{-}H}$ = 8.4 Hz, 2 H, H^6), 4.79 (broad s, 3 H, $^+$N-CH$_3$), 1.38 (s, 18 H, ^{t}Bu). ^{13}C{^{1}H} NMR (acetone-d^6, 75.48 MHz): δ 173.3 (s, C^9), 165.3 (s, $C^{3/4}$), 160.4 (s, $C^{3/4}$), 142.7 (s, C^7), 142.7 (s, C^2), 131.8 (s, C^8), 129.0 (s, C^5), 126.5 (s, C^6), 51.9 (s, $^+$N-CH$_3$), 36.7 (s, CMe$_3$), 31.2 (s, CMe_3).

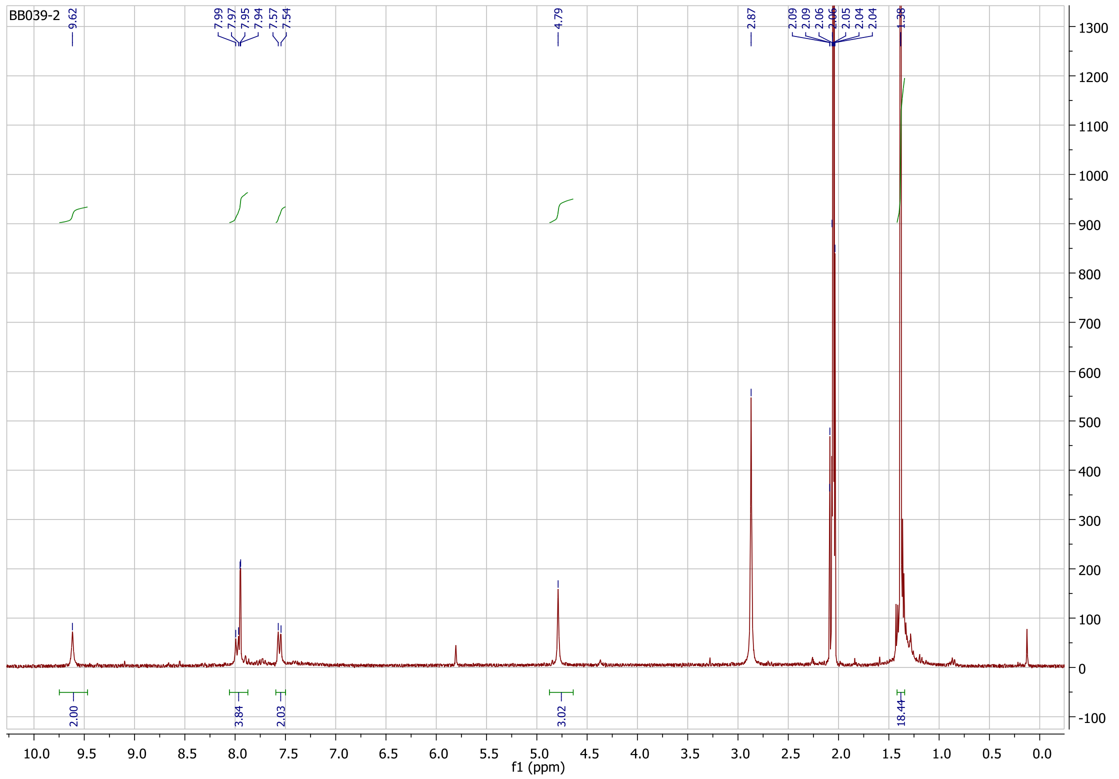

Figure S13. ^{1}H NMR spectrum of **7**.

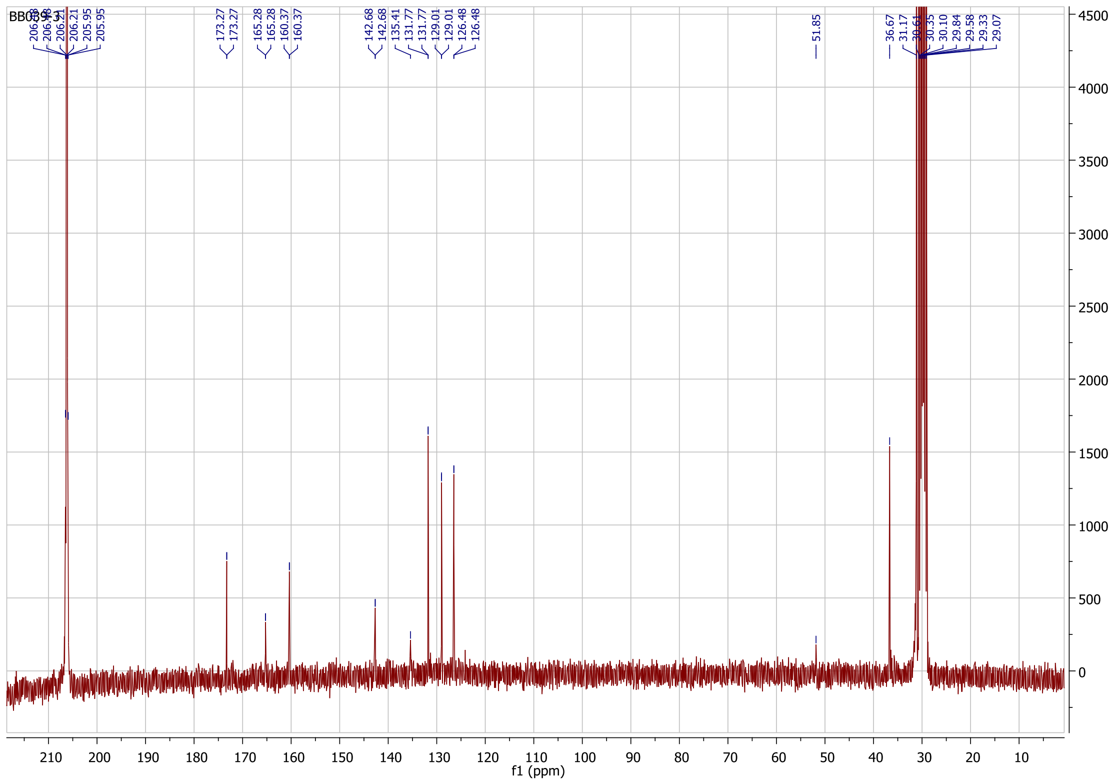

Figure S14. ^{13}C NMR spectrum of **7**.

S2. X-ray crystallography

Crystals of each sample were mounted in MiTeGen MicroMesh systems and fixed in the cold nitrogen stream on a diffractometer. Diffraction intensities were recorded al low temperature on an Oxford Diffraction Xcalibur-3/Sapphire3-CCD diffractometer, equipped with Mo-Kα radiation and graphite monochromator or Rigaku HG Saturn724+ (2×2 bin mode). Data were processed using the CrystAlisPro-CCD and –RED software or CrystalClear-SM Expert 3.1 b27, been the absorption correction done at this stage.[S2]

The structures of all samples were determined by the direct methods routines with SHELXS or SHELXT programs and refined by full-matrix least-squares methods on F^2 in SHELXL.[S3] Non-hydrogen atoms were generally refined with anisotropic thermal parameters. Hydrogen atoms were included in idealised positions. No missed symmetry was reported by PLATON.[S4]

Refinement results are included in Table S1. Computer programs used in this analysis were run through WinGX.[S5] Scattering factors for neutral atoms were taken from reference S6.

Crystallographic details for (C^N^{pz}^C)AuCl (2): Orange block (0.1×0.1×0.3 mm) grown by slow evaporation of a saturated solution of the crude in a mixture DMSO:acetone (1:9) at room temperature. The asymmetric unit is formed by one half molecule of **2**. A positional disorder was found in one of the ^{t}Bu groups and this was modelled with 0.6 and 0.4 occupancy. Some peaks higher than 1 e.Å^{-3} were found in the vicinity of the Au center without any chemical meaning.

(C^N^{pz}^C)Audmpz (4): Yellow needles (0.21×0.03×0.01 mm) grown by slow evaporation of a concentrated solution in dichloromethane. Several peaks higher than 1 e.Å^{-3} were found in the vicinity of the Au center without any chemical meaning.

(C^N^{pz}^C)AuC≡CPh (5a): Yellow needles (0.2×0.05×0.05 mm) grown by slow evaporation of a solution of the complex in a mixture CH_2Cl_2:*i*-PrOH (9:1) at room temperature. Several peaks higher than 1 e.Å^{-3} were found in the vicinity of the Au center without any chemical meaning.

(C^N^{pz}^C)AuC≡C^{t}Bu (5b): Yellow plate (0.16×0.11×0.07 mm) grown by slow evaporation of a saturated solution of the complex in a mixture CH_2Cl_2:*i*-PrOH (9:1) at room temperature. Several peaks higher than 1 e.Å^{-3} were found in the vicinity of the Au center without any chemical meaning.

Table S1. Selected crystal data and structure refinement details for **2**, **4**, **5a** and **5b**.

	(C^N^{pz}^C)AuCl **(2)**	(C^N^{pz}^C)Audmpz **(4)**	(C^N^{pz}^C)AuC≡CPh **(5a)**	(C^N^{pz}^C)AuC≡C^{t}Bu **(5b)**
Empirical formula	$C_{24}H_{26}AuClN_2$	$C_{29}H_{33}AuN_4$	$C_{32}H_{31}AuN_2$	$C_{30}H_{35}AuN_2$
F_w	574.88	634.56	640.55	620.56
T (K)	140(2)	100(2)	140(2)	100(2)
crystal system, space group	orthorhombic; P b c n	monoclinic; $P2_1/c$	Monoclinic; $P2_1/c$	Monoclinic, $P2_1/c$
a(Å)	15.6786(6)	10.4846(7)	8.4040(3)	12.3700(9)
b(Å)	18.9401(7)	21.4472(15)	13.1184(4)	12.3194(9)
c(Å)	7.1383(3)	11.2718(8)	23.6000(7)	18.1788(13)
α(deg)	90	90	90	90
β(deg)	90	94.619(2)	90	104.680(13)
γ(deg)	90	90	90	90
volume (A^3)	2119.75(14)	2526.4(3)	2601.83(14)	2679.9(4)
Z	4	4	4	4
D_{calcd} (Mg/m^3)	1.801	1.668	1.635	1.538
absorption coefficient (mm^{-1})	7.078	5.847	5.677	5.509
F(000)	1120	1256	1264	1232
θ range for data collection (º)	3.315 to 32.741	2.626 to 27.503	2.8727 to 29.9208	2.444 to 27.678
data // restraints // params	3713 // 0 // 140	5760 // 0 // 315	7043 // 0 // 322	6151 // 0 // 307
goodness-of-fit on F^2[a]	1.093	1.012	1.263	1.037
final R indexes [I>2σ(I)][a]	R1 = 0.0286, wR2 = 0.0648	R1 = 0.0247, wR2 = 0.0596	R1 = 0.0424, wR2 = 0.0974	R1 = 0.0279, wR2 = 0.072
R indexes (all data)[a]	R1 = 0.0395, wR2 = 0.0705	R1 = 0.0295, wR2 = 0.0622	R1 = 0.0481, wR2 = 0.0999	R1 = 0.031, wR2 = 0.0748
largest diff peak and hole (e.Å $^{-3}$)	2.324 and -2.686	1.359 and -0.883	2.134 and -3.982	1.552 and -1.518

[a] $R1 = \Sigma(|F_o| - |F_c|)/\Sigma|F_o|$; $wR2 = [\Sigma w(F_o^2 - F_c^2)^2/\Sigma wF_o^2]^{1/2}$; goodness of fit = $\{\Sigma[w(F_o^2 - F_c^2)^2]/(N_{obs} - N_{param})\}^{1/2}$; $w = [\sigma^2(F_o) + (g_1P)^2 + g_2P]^{-1}$; $P = [\max(F_o^2;0 + 2F_c^2]/3$.

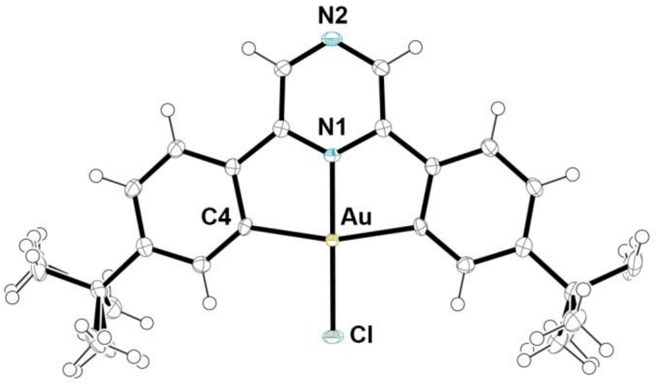

Figure S15: Structure of (C^N^{pz}^C)AuCl (**2**). Selected bond distances (Å) and angles (°): Au-N1 1.972(3), Au-Cl 2.2651(11), Au-C4 2.075(3), N1-Au-C4 81.28(8), C4-Au-Cl 98.72(8), C4-Au-C4’ 162.56(16).

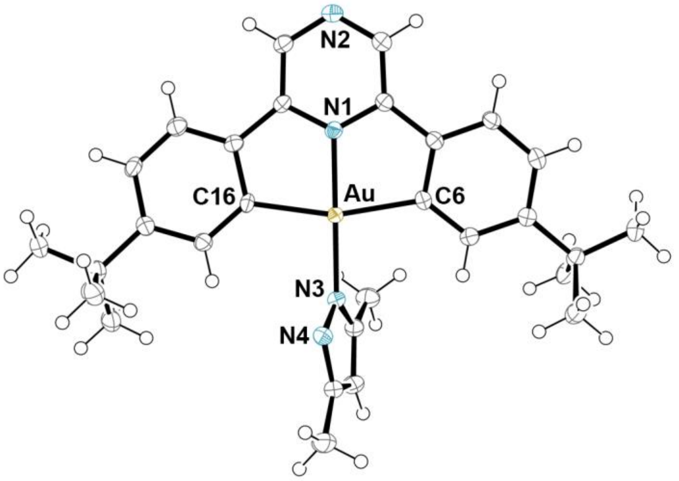

Figure S16: Structure of (C^N^{pz}^C)Au-dmpz (**4**). Selected bond distances (Å) and angles (º): Au-N1 1.979(2), Au-C6 2.080(3), Au-C16 2.073(3), Au-N3 1.991(2), C6-Au-N1 81.39(10), C16-Au-N1 81.32(11), C6-Au-N3 98.88(10), C16-Au-N3 98.38(11), N1-Au-N3 178.33(9).

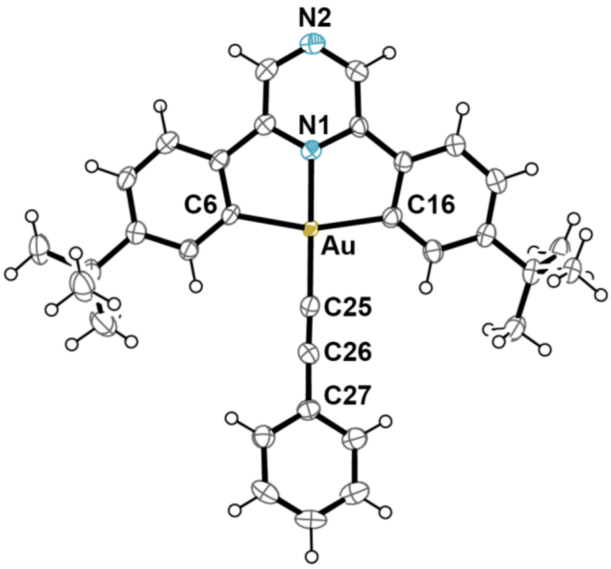

Figure S17: Structure of (C^N^{pz}^C)AuC≡CPh (**5a**). Selected bond distances (Å) and angles (°): Au1 N1 1.997(5), Au-C6 2.070(6), Au-C16 2.083(7), Au-C25 1.979(7), C25-C26 1.199(9), N1-Au-C6 80.4(2), N1-Au-C16 81.2(2), C6-Au-C25 99.3(3), C16-Au-C25 99.0(3), C6-Au-C16 161.6(3), Au-C25-C26 175.9(6), C25-C26-C27 177.4(7).

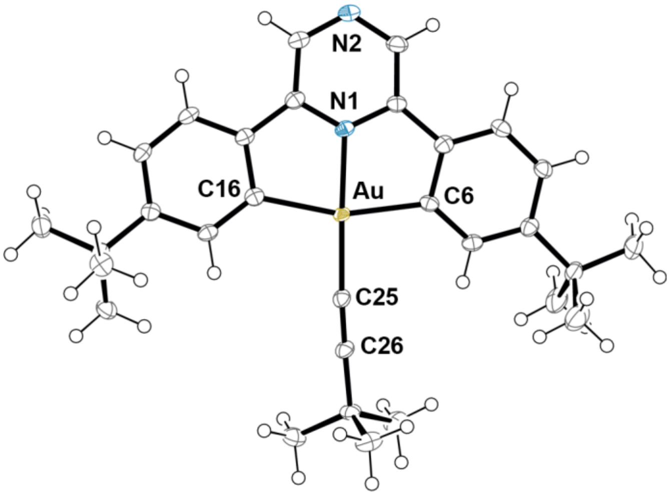

Figure S18: Structure of (C^N^{pz}^C)AuC≡C^{t}Bu **(5b)**. Selected bond distances (Å) and angles (°): Au1 N1 2.004(2), Au-C6 2.073(3), Au-C16 2.087(3), Au1-C25 1.971(3), C25-C26 1.202(4), N1-Au-C6 80.85(11), N1-Au-C16 80.64(11), C6-Au-C25 97.13(11), C16-Au-C25 101.38(11), C6-Au-C16 161.48(12), Au-C25-C26 175.8(3), C25-C26-C27 177.2(3).

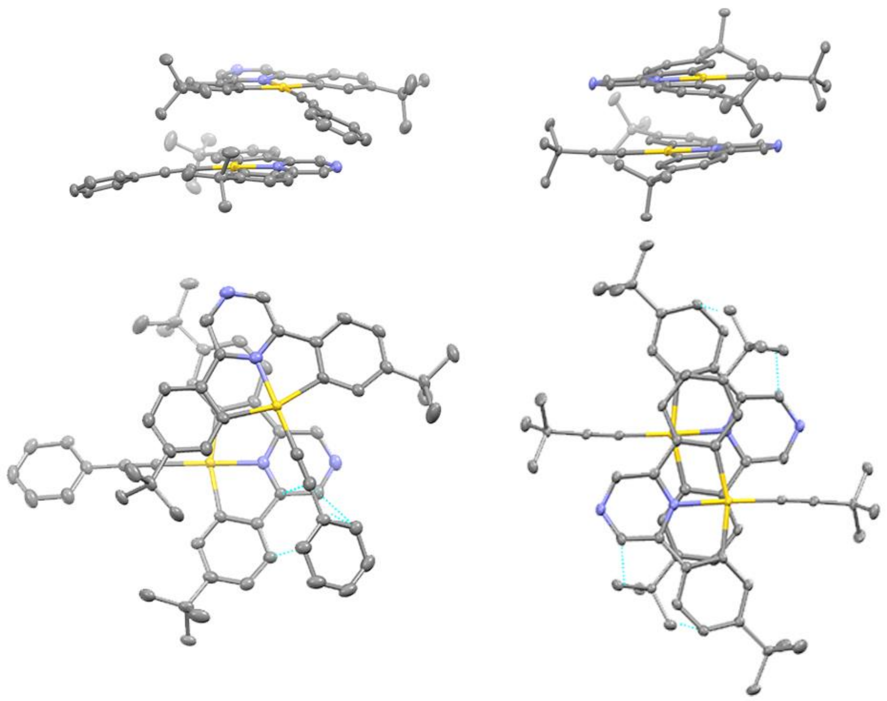

Figure S19. Left: Analysis of the crystal network of complex (C^N^{pz}^C)AuC≡CPh (**5a**), showing the presence of π···π interactions between the phenyl ring of the alkynyl of one molecule, and the C^N^{pz}^C of the next molecule. Right: Head-to-tail arrangement in (C^N^{pz}^C)AuC≡C^{t}Bu (**5b**); the most relevant interactions are CH(^{t}Bu)··· C^N^{pz}^C between neighbouring molecules.

S3. Photophysical Properties

UV−visible absorption spectra were recorded using a Perkin-Elmer Lambda 35 UV/vis spectrometer. Excitation and emission spectra were measured in a (TCSPC) FluoroLog Horiba Jobin Yvon spectrofluorometer. Lifetime measurements were done with a Datastation HUB-B with a nanoLED controller and software DAS6. The nanoLEDs employed for lifetime measurements were of 370 nm with pulse lengths of 0.8−1.4 ns. Quantum yields in the solid state were measured upon excitation at 370-400 nm using an F-3018 integrating sphere mounted on a Fluorolog 3-11 Tau-3 spectrofluorimeter. In solution, relative quantum yields were determined with reference to $[Ru(bpy)_3]^{2+}$ in H_2O (ϕ_{stn} = 2.8 %) or versus Quinine sulfate in H_2SO_4 0.05 M (ϕ_{stn} = 51 %) in accordance with ref. S7.

Table S2. Absorption data for complexes **2-9** in CH_2Cl_2 (10^{-5} M)

Complex	Absorbance [nm] (10^3 ε/M^{-1} cm^{-1})
**2**	278(37.7), 295(22.3), 324(16.2), 349(9.6), 407(3.6), 430(5.7), 455(6.2).
**3**	259(32.8), 281(18.5), 296(15.4), 321(10.4), 349(6.6), 407(3.5), 431(5.7), 455(5.8).
**4**	318(15.0), 346(8.4), 408(2.4), 430(4.3), 454(4.5).
**5a**	289(20.5), 322(16.3), 340(14.3), 404(5.4), 423(7.0), 447(6.6).
**5b**	321 (17.5), 350(12.8), 406(6.0), 425(7.2), 440*sh*(3.2)

Table S3. PL properties of the complexes in the solid state.

	λ_{em} / nm (λ_{ex} / nm) {ϕ / %}[a)]	
Complex	298 K	77 K
**2**	[b)]	495*br*, 560_{max}, 580*sh*, 603, 624*sh*, 650*sh* (300-450)
**3**	507*sh*, 535_{max}, 548*sh*, 572 (300-465) {2.9}	564_{max}, 580*sh* (300-460)
**4**	490*sh*, 531_{max}, 564*br* (300-460)	548_{max}, 580*sh* (300-470)
**5a**	489*sh*, 523_{max}, 538*sh*, 556, 588 (300-460) {4.5}	530_{max}, 550*sh* (300-450)
**5b**	523_{max}, 550, 591*sh* (300-460) {8.3}	532_{max}, 582*sh* (300-470)
**7**	623, 680*sh* (320-500)	670*br* (350-500)

a) Determined by absolute methods using an integrating sphere. b) Non-emissive.

Table S4. PL properties of the complexes in solution.

Complex	Medium (T/K)	λ_{em} / nm (λ_{ex} / nm)	τ / *ns*[a)]	{ϕ / %}[b)] (k_r ; k_{nr} / s^{-1})[c)]
**2**	CH_2Cl_2 (298)[e)]	482-530*br* (384, 403, 410sh, 430, 480)		
	CH_2Cl_2 (77)[e)]	488*sh*, 508, 534_{max} (300-420)		
	CH_2Cl_2 10^{-5} M + HNB_2[b)] (298)	458_{max} (300-420)		
	CH_2Cl_2 10^{-5} M + HNB_2[b)] (77)	437, 497_{max}, 524*sh* (300-415)	3.04±0.02	
**3**	CH_2Cl_2 (298)[e)]	477*sh*, 536_{max}, 562*sh* (318, 348, 407, 429, 454)	5.96±0.05	0.184 ($k_r = 3.78 \times 10^5$ s^{-1}; $k_{nr} = 1.68 \times 10^8$ s^{-1}).
	CH_2Cl_2 (77)[e)]	550_{max}, 570*sh* (300-400)		
	MeOH (298)	483*sh*, 543_{max}, 570*sh* (338br, 402-460)		
**4**	CH_2Cl_2 (298)[e)]	495sh, 572_{max} (380, 400)	15.4±0.1 (81%), 115.2±10.8 (19%).	0.008 ($k_r = 4.15 \times 10^3$ s^{-1}; $k_{nr} = 5.4 \times 10^7$ s^{-1}).
	CH_2Cl_2 (77)[e)]	537_{max}, 566*sh* (300-400)		
**5a**	CH_2Cl_2 (298)[e)]	526_{max}, 558*sh*, 599*sh* (307, 359, 399, 419, 444)	161±7 (53%), 6.5±0.1 (47%).	0.462 ($k_r = 3.29 \times 10^5$ s^{-1}; $k_{nr} = 7.11 \times 10^7$ s^{-1}).
	CH_2Cl_2 (77)[e)]	525_{max}, 556*sh*, 600*sh* (300-420)		
	CH_2Cl_2 10^{-5} M + HTfO (298)	450_{max}, 473*sh*, 495*sh* (310-420)	5.27±0.01	2.8 ($k_r = 5.31 \times 10^6$ s^{-1}; $k_{nr} = 1.95 \times 10^8$ s^{-1})
	MeOH 10^{-4} M (298)	476*br*, 535_{max}, 564*sh* (300-425)		
	MeOH 10^{-4} M (77)	536_{max}, 568*sh* (300-400)		
**5b**	CH_2Cl_2 (298)[e)]	473*sh*, 525_{max}, 554*sh*, 596*sh* (373, 403*sh*, 431, 459).	130±2 (48%), 5.0±0.3 (52%).	0.512 ($k_r = 7.8 \times 10^4$ s^{-1}; $k_{nr} = 1.55 \times 10^7$ s^{-1})
	CH_2Cl_2 (77)[e)]	522_{max}, 560*sh*, (300-420).		
**7**	CH_2Cl_2 10^{-4} M (298)	460 (360-400)	3.46±0.01(83%), 10.63±0.05 (17%)	3.75($k_r = 9.60 \times 10^6$ s^{-1}; $k_{nr} = 2.66 \times 10^8$ s^{-1})
	CH_2Cl_2 10^{-4} M (77)	650 (320-450)		

a) Measured at λ_{max}^{em} b) Relative to standard.[S7] c) $k_r = \phi / \tau_{av}$; $k_{nr} = 1 / [\tau_{av} \times (1-\phi)]$. d) HNB_2 refers to $[H(OEt_2)_2][H_2N\{B(C_6F_5)_3\}_2]$. e) The same emission was monitored at several concentrations (range 10^{-3} M to 10^{-5} M) and in different solvents (typically THFMe-2, Toluene and Et_2O).

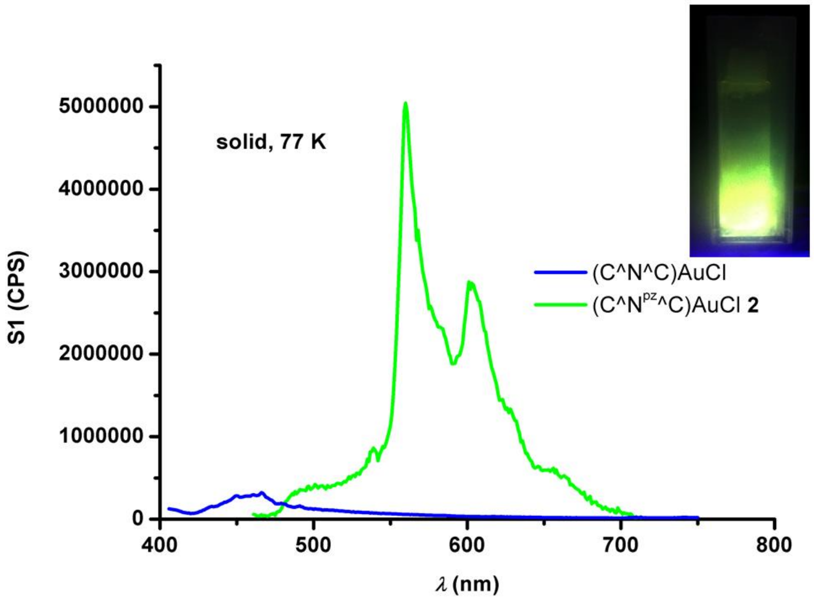

Figure S20. Comparison between the emission spectra of (C^N^{py}^C)AuCl (—) and (C^N^{pz}^C)AuCl (—) in the solid state at 77 K. Inset: Photograph of complex **2** under UV light (365 nm) in the solid state at 77 K.

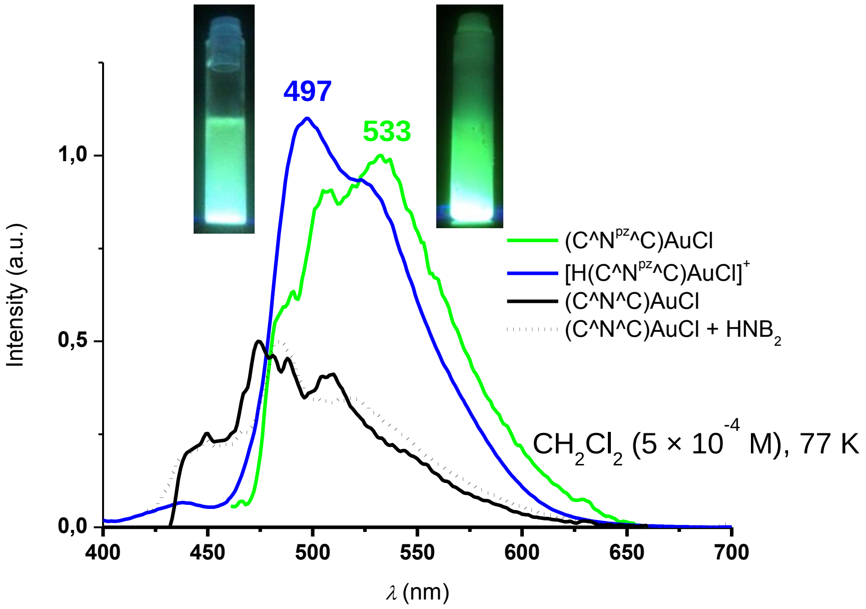

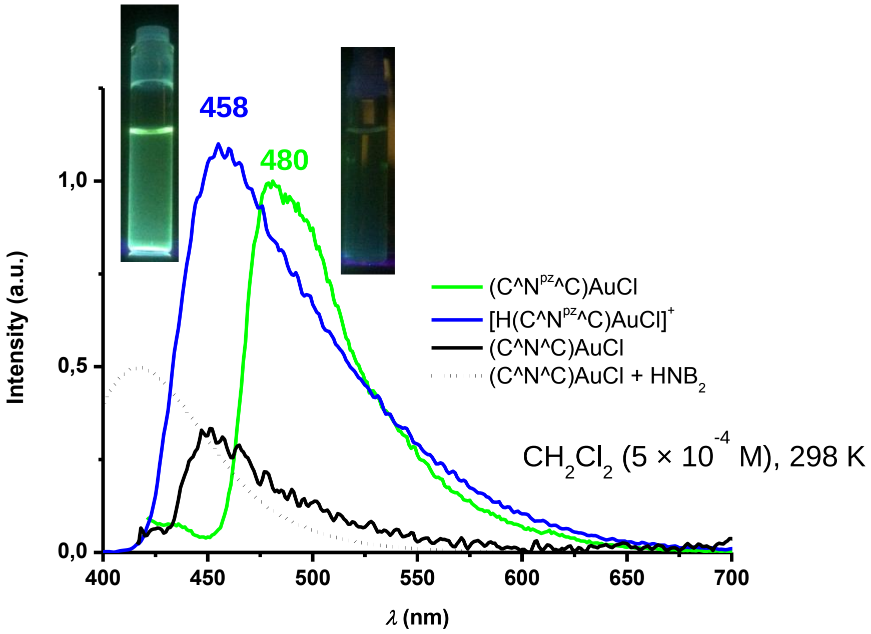

Figure S21. Photoluminescence of **2** in CH_2Cl_2 in the absence (green line) and presence (blue) of $[H(OEt_2)_2][H_2N\{B(C_6F_5)_3\}_2]$.

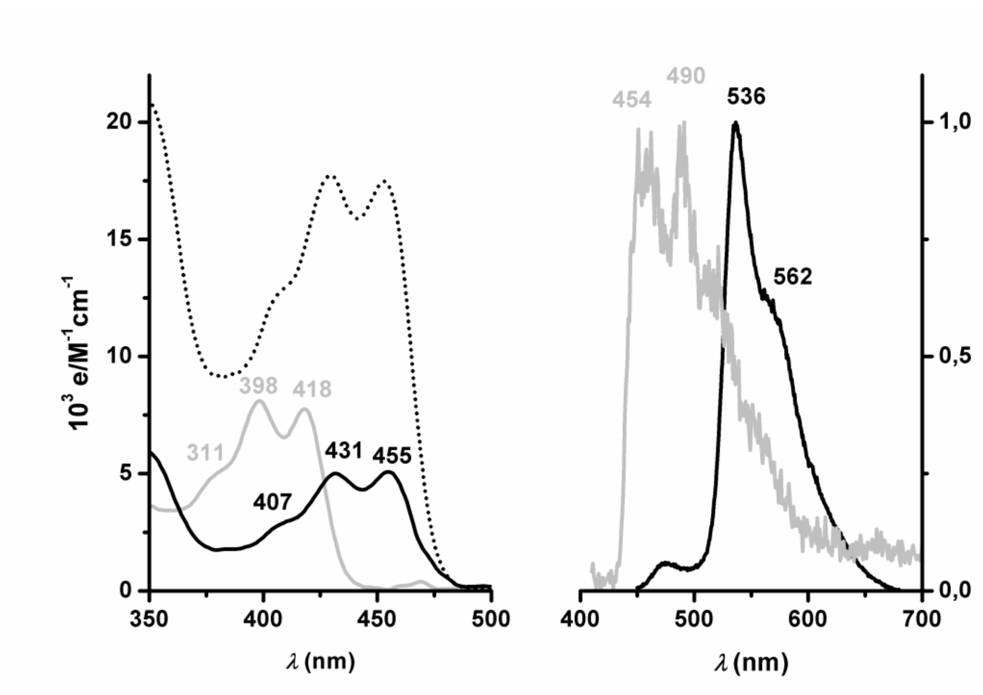

Figure S22. Overlay of the UV-Vis spectra (left) and the emission band (right) of complexes (C^N^{py}^C)AuCN (grey lines) and (C^N^{pz}^C)AuCN **3** (black solid lines) in CH_2Cl_2 (1×10^{-5} M) solution at room temperature. The excitation band (a.u.) of **3** (black dotted line) mimics the absorption band.

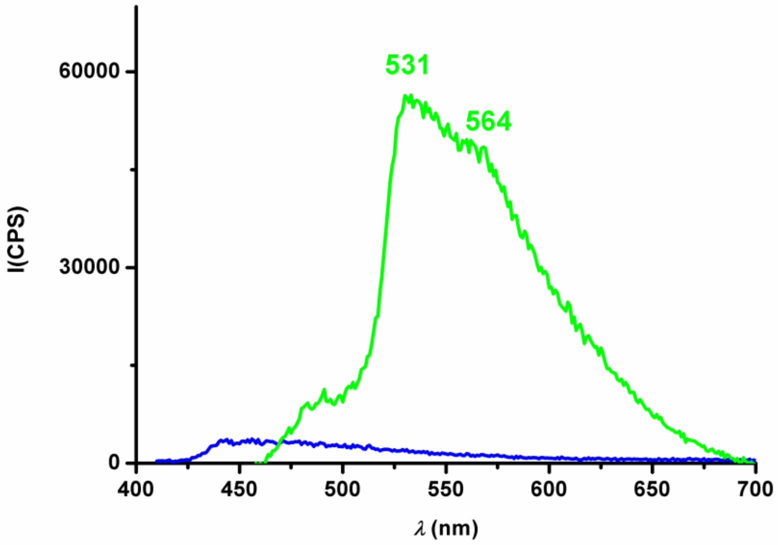

Figure S23. Comparison between the emission spectra of (C^N^{py}^C)Audmpz (—) and (C^N^{pz}^C)Audmpz (—) in the solid state at 298 K.

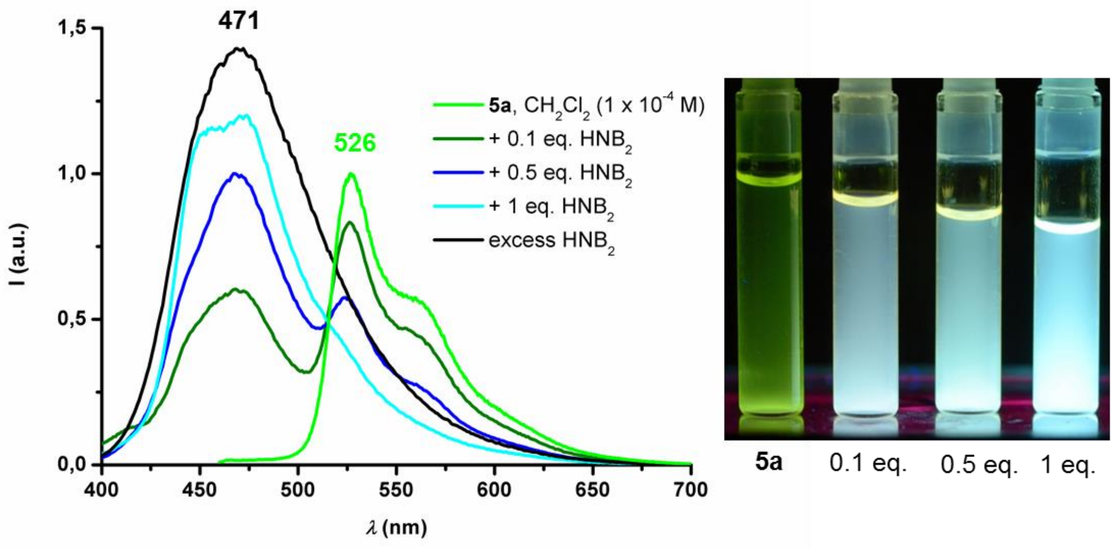

Figure S24. Photoluminescence of **5a** before and after adding $[H(OEt_2)_2]^+[H_2N\{B(C_6F_5)_3\}_2]^-$, CH_2Cl_2, 298 K. Left to right: 0, 0.1, 0.5, 1 equiv of H^+. Photographs under UV light (365 nm).

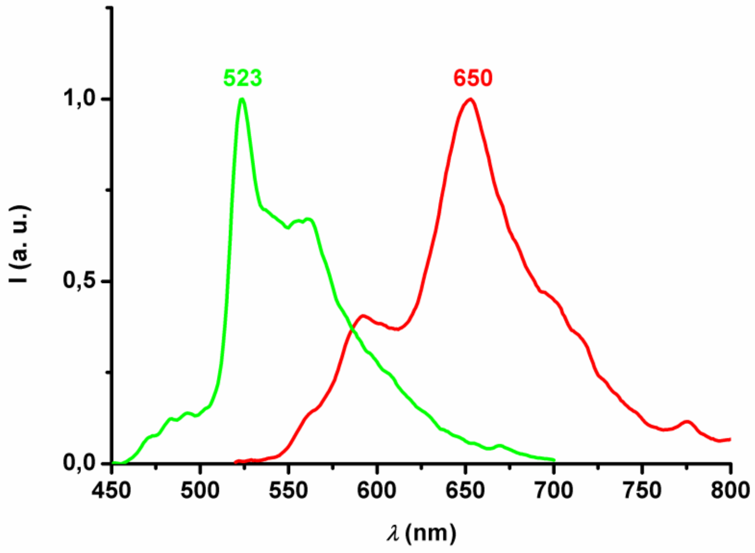

Figure S25. Photoluminescence of **5a** and **5a** + 1 equiv. of $[H(OEt_2)_2]^+[H_2N\{B(C_6F_5)_3\}_2]^-$ in the solid state.

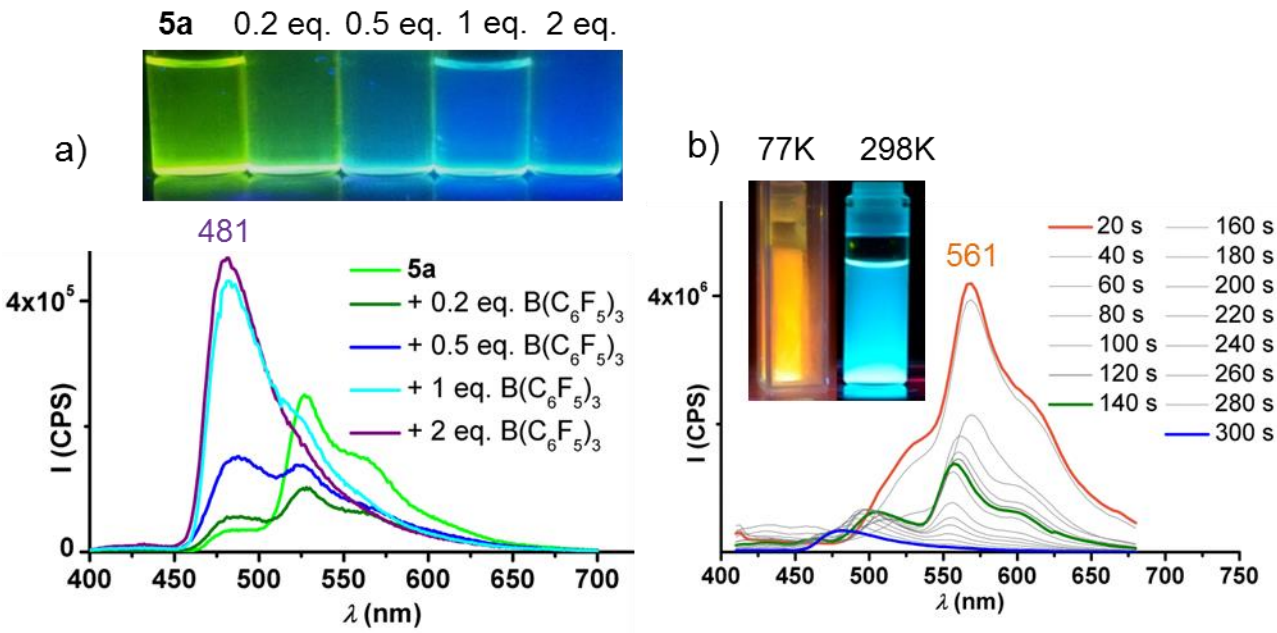

Figure S26. (a) PL response of **5a** (CH_2Cl_2, 1 × 10^{-4} M) to the addition of $B(C_6F_5)_3$ at 298 K. (b) PL spectra of a solution of **5a** + 1 $B(C_6F_5)_3$ (CH_2Cl_2, 1 × 10^{-4} M) upon warming from 77 K to 298 K (the times given merely serve to illustrate the spectral changes during the warming-up process).

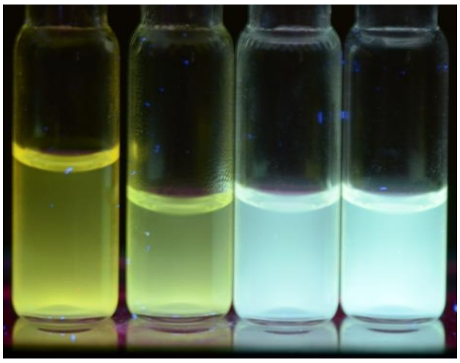

Figure S27. Pictures of the Photoluminescence of **5a** before and after adding $ZnCl_2$, CH_2Cl_2, 298 K. Left to right: 0, 0.1, 0.5, 1 equiv of $ZnCl_2$. Photographs under UV light (365 nm).

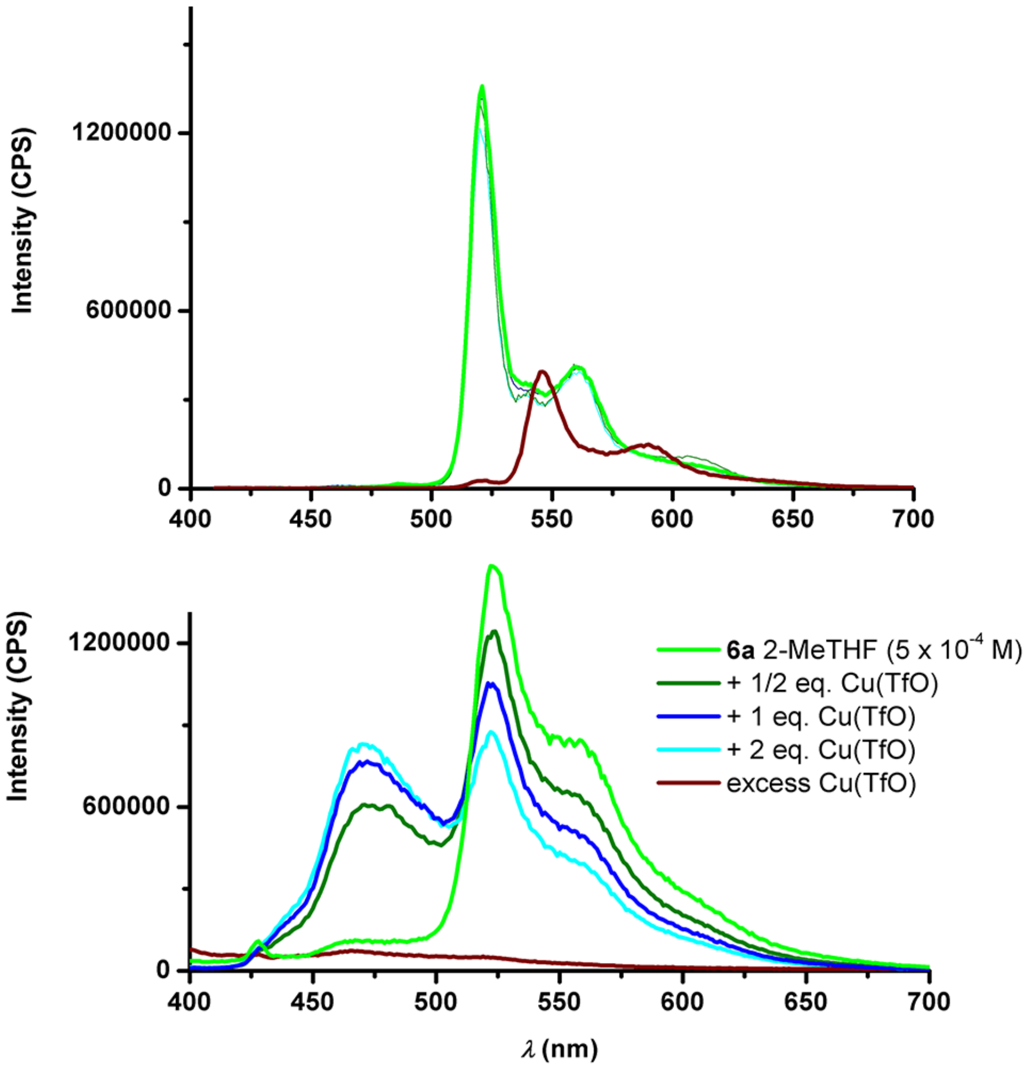

Figure S28. PL response of **5a** (2-MeTHF, 5 × 10^{-4} M) to the addition of CuOTf at 77 K (top) and 298 K (bottom).

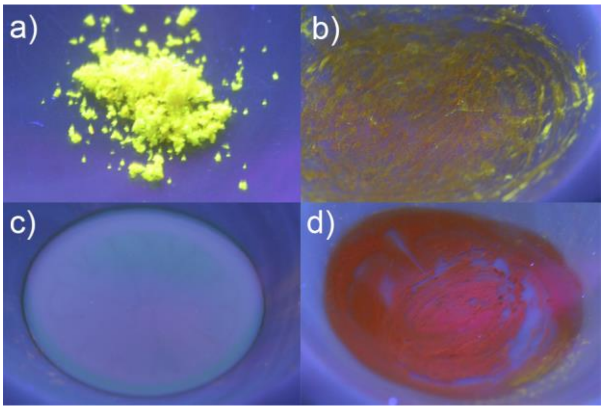

Figure S29. Photographs under UV light (365 nm) of (a) Complex **5b**. (b) Complex **5b** + $[H(OEt_2)_2]^+[H_2N\{B(C_6F_5)_3\}_2]^-$ after grinding. (c) The same mixture dissolved in 5 mL of CH_2Cl_2. (d) Residue obtained after evaporation of the solvent.

S4. Theoretical Calculations

The structure of the ground (S^0) and lowest triplet (T^1) states for all of the complexes were optimised at DFT(PBE0)[S8] level using the ORCA quantum chemistry program.[S9] During the optimisation a def2-SVP basis set[S10] was used for all of the atoms. The excited state energies were computed using linear response time-dependent density functional theory (LR-TDDFT)[S11] within the Tamm-Damcoff Approximation.[S12] These were simulated using the optimally tuned[S13] range-separated functional (LC-BLYP).[S14] In range-separated functionals the amount of exact exchange is weighted according to the inter electron distance, r_{12}:

$$\frac{1}{r_{12}} = erfc(\mu \cdot \mathrm{r}_{12}) \cdot \mathrm{r}_{12}^{-1} + erf(\mu \cdot \mathrm{r}_{12}) \cdot \mathrm{r}_{12}^{-1}$$

where $\mathrm{erf}(x) = \frac{2}{\sqrt{x}}\int_0^x \exp(-t^2)\,dt$

and $\mathrm{erfc(x)} = 1 - \mathrm{erf(x)}$.

The optimal value of μ was obtained using the ΔSCF method.[S13] Here we minimised the energy difference between energy of the HOMO (ε_{HOMO}) and first ionisation potential (IP) of the neutral system and the energy difference between energy of the HOMO of the anionic system, (ε_{HOMO+1}) and the electron affinity (EA) of the neutral system. These conditions are expressed as:

$$J_0 = \left|\varepsilon^{\mu}_{HOMO}(N) + IP^{\mu}(N)\right|$$

and

$$J_1 = \left|\varepsilon^{\mu}_{HOMO}(N+1) + EA^{\mu}(N)\right|$$

Therefore our overall goal is to minimise the relationship:

$$J(\mu) = J_0(\mu) + J_1(\mu)$$

Optimisation of the range-separated parameter, μ, using the above expression was performed within the approximation of the LC-BLYP exchange-correlation functional. The effect of the solvent was included using the PCM approach and the parameters of dichloromethane. This was only included during the calculation of the excited states and not during the tuning of μ. This is consistent with the conclusions of refs S15 who reported that μ tuning within a continuum solvent model can result in unrealistic values due to a significant underestimation of vertical ionisation potential. For all of the complexes studied herein, he optimum value of μ, was found to be a consistent value of μ=0.18±0.01 bohr^{-1} similar to a study of iridium photosensitisers.

Table S5. Selected bonds [Å] and angles [º] for the computed structures of **2**, **3**, **4** and **5a.**

	**2**		**3**		**4**		**5a**	
	S^0	T^1	S^0	T^1	S^0	T^1	S^0	T^1
Au-N (Å)	1.97	1.96	2.01	1.98	1.99	1.99	2.03	1.97
Au-C(C^N^{pz}^C) (Å)	2.07	2.07	2.10	2.08	2.12	2.10	2.11	2.07
Au-X[a)] (Å)	2.29	2.30	1.96	1.97	2.01	2.00	1.96	1.97
N-Au-X[a)] (º)	179	178	180	180	171	175	177	176
N-Au-C(C^N^{pz}^C) (º)	82	80	81	80	80	80	80	81

a) X refers to the atom belonging to the ancillary ligand that is directly bonded to Au (X = Cl 2, *C*≡N 3, N 4, *C*≡CPh)

Table S6: Molecular Orbital Composition for singlet and triplet in **2**.

	S^1			T^1		
	Au	Cl	C^N^{pz}^C	Au	Cl	C^N^{pz}^C
L+1	0.5	0.0	99.5	42.3 $^{\alpha}$ 13.9 $^{\beta}$	16.1$^{\alpha}$ 1.5 $^{\beta}$	41.5 $^{\alpha}$ 84.6 $^{\beta}$
LUMO	9.3	1.1	89..7	3.0 $^{\alpha}$ 2.8 $^{\beta}$	0.3 $^{\alpha}$ 0.1$^{\beta}$	96.7 $^{\alpha}$ 97.1 $^{\beta}$
HOMO	10.5	4.1	85.4	6.8 $^{\alpha}$ 6.9 $^{\beta}$	1.2 $^{\alpha}$ 6.6. $^{\beta}$	92.0 $^{\alpha}$ 86.5 $^{\beta}$
H-1	14.5	13.0	72.5	19.6 $^{\alpha}$ 16.5 $^{\beta}$	21.7 $^{\alpha}$ 17.6 $^{\beta}$	58.6 $^{\alpha}$ 65.9 $^{\beta}$
H-2	6.6	0.4	92.9	5.5 $^{\alpha}$ 9.4 $^{\beta}$	2.9 $^{\alpha}$ 2.0 $^{\beta}$	91.5 $^{\alpha}$ 88.6 $^{\beta}$
H-3	2.9	0.7	71.8	9.6 $^{\alpha}$ 12.4 $^{\beta}$	10.0 $^{\alpha}$ 38.2$^{\beta}$	80.4 $^{\alpha}$ 49.4$^{\beta}$

Table S6 (Continued): Molecular Orbital Composition for singlet and triplet in (C^N^{py}^C)AuCl.

	S^1			T^1		
	Au	Cl	C^N^{py}^C	Au	Cl	C^N^{py}^C
L+1	0.3	0.0	99.7	43.0 $^{\alpha}$ 14.0 $^{\beta}$	17.4 $^{\alpha}$ 1.3 $^{\beta}$	39.6 $^{\alpha}$ 84.7 $^{\beta}$
LUMO	10.2	1.1	88.6	5.2 $^{\alpha}$ 3.2$^{\beta}$	0.4 $^{\alpha}$ 0.1 $^{\beta}$	94.4 $^{\alpha}$ 96.7 $^{\beta}$
HOMO	4.5	0.2	95.4	5.7 $^{\alpha}$ 19.9$^{\beta}$	0..9 $^{\alpha}$ 17.7 $^{\beta}$	93.4 $^{\alpha}$ 62.5 $^{\beta}$
H-1	20.0	16.6	63.3	10.1 $^{\alpha}$ 2.2 $^{\beta}$	4.9 $^{\alpha}$ 1.0 $^{\beta}$	84.9 $^{\alpha}$ 96.9 $^{\beta}$
H-2	8.8	0.0	91.2	14.3 $^{\alpha}$ 9.9 $^{\beta}$	15.4 $^{\alpha}$ 2.0 $^{\beta}$	70.2 $^{\alpha}$ 88.1 $^{\beta}$
H-3	2.8	3.4	93.8	9.0 $^{\alpha}$ 11.8 $^{\beta}$	8.6 $^{\alpha}$ 39.9 $^{\beta}$	82.4 $^{\alpha}$ 48.3 $^{\beta}$

Table S7: Molecular Orbital Composition for singlet and triplet in **3**.

	S^1			T^1		
	Au	C≡N	C^N^{pz}^C	Au	C≡N	C^N^{pz}^C
L+1	0.4	0.0	99.6	15.6^{α} 16.5^{β}	3.1^{α} 3.5^{β}	81.2^{α} 80.0^{β}
LUMO	11.0	2.4	86.6	1.0^{α} 3.2^{β}	0.1^{α} 0.0^{β}	98.9^{α} 96.8^{β}
HOMO	3.0	0.0	97.0	9.0^{α} 1.3^{β}	1.6^{α} 0.1^{β}	89.4^{α} 98.6^{β}
H-1	20.2	6.2	73.6	4.8^{α} 21.1^{β}	0.0^{α} 7.6^{β}	95.2^{α} 71.3^{β}
H-2	5.9	0.1	94.0	20.9^{α} 8.8^{β}	7.9^{α} 0.1^{β}	71.2^{α} 91.1^{β}
H-3	3.0	0.1	96.9	6.3^{α} 1.1^{β}	0.7^{α} 0.8^{β}	93.0^{α} 98.1^{β}

Table S7 (Continued): Molecular Orbital Composition for singlet and triplet in (C^N^{py}^C)AuCN.

	S^1			T^1		
	Au	C≡N	C^N^{py}^C	Au	C≡N	C^N^{py}^C
L+1	0.3	0.0	99.7	18.1^{α} 19.9^{β}	7.5^{α} 2.4^{β}	64.6^{α} 77.7^{β}
LUMO	12.8	2.9	84.3	1.9^{α} 21.8^{β}	0.1^{α} 29.1^{β}	99.1^{α} 49.1^{β}
HOMO	2.9	0.0	97.1	9.8^{α} 3.0^{β}	3.6^{α} 25.7^{β}	75.2^{α} 71.3^{β}
H-1	20.0	5.9	74.1	4.5^{α} 5.8^{β}	2.1^{α} 44.4^{β}	92.7^{α} 49.8^{β}
H-2	8.6	0.1	91.4	20.7^{α} 2.0^{β}	62.2^{α} 4.9^{β}	27.8^{α} 93.1^{β}
H-3	0.8	0.1	99.0	7.7^{α} 2.5^{β}	79.2^{α} 79.3^{β}	16.0^{α} 18.2^{β}

Table S8: Molecular Orbital Composition for singlet and triplet in **4**.

	S^1			T^1		
	Au	dmpz	C^N^{pz}^C	Au	dmpz	C^N^{pz}^C
L+1	2.6	0.1	97.3	41.4^{α} 17.4^{β}	10.7^{α} 1.5^{β}	$47.8.^{\alpha}$ 81.1^{β}
LUMO	10.7	0.8	88.5	2.9^{α} 19.6^{β}	0.1^{α} 42.1^{β}	97.0^{α} 38.3^{β}
HOMO	11.4	70.6	18.0	11.9^{α} 3.8^{β}	1.1^{α} 9.4^{β}	87.0^{α} 86.8^{β}
H-1	1.2	95.6	3.3	5.1^{α} 10.7^{β}	0.2^{α} 45.0^{β}	94.7^{α} 44.4^{β}
H-2	4.9	0.7	94.3	17.8^{α} 3.2^{β}	38.5^{α} 0.5^{β}	43.7^{α} 96.3^{β}
H-3	9.0	31.3	59.8	5.7^{α} 5.8^{β}	1.0^{α} 0.8^{β}	93.3^{α} 93.4^{β}

Table S8 (Continued): Molecular Orbital Composition for singlet and triplet in (C^N^{py}^C)Audmpz.

	S^1			T^1		
	Au	dmpz	C^N^{py}^C	Au	dmpz	C^N^{py}^C
L+1	0.3	0.1	99.5	27.9^{α} 16.9^{β}	3.7^{α} 3.6^{β}	78.2^{α} 79.6^{β}
LUMO	11.1	1.0	87.9	0.7^{α} 3.0^{β}	0.3^{α} 0.0^{β}	97.9^{α} 97.0^{β}
HOMO	9.3	75.0	15.8	21.2^{α} 1.3^{β}	1.8^{α} 0.1^{β}	88.4^{α} 98.6^{β}
H-1	2.0	93.2	4.8	5.2^{α} 20.8^{β}	0.0^{α} 7.3^{β}	95.5^{α} 71.9^{β}
H-2	3.6	1.1	95.3	10.1^{α} 9.5^{β}	7.5^{α} 0.0^{β}	71.8^{α} 90.5^{β}
H-3	12.5	23.1	64.5	4.8^{α} 2.4^{β}	0.6^{α} 2.4^{β}	91.7^{α} 95.1^{β}

Table S9: Calculated emission for complexes **2**, **3**, **4** and **5a** compared with the corresponding pyridine analogous in CH_2Cl_2.

Complex	λ_{em} / nm (T^1 state)
(C^N^{pz}^C)AuCl **2**	540.9
(C^N^{py}^C)AuCl	513.2
(C^N^{pz}^C)AuCN **3**	527.4
(C^N^{py}^C)AuCN	476.8
(C^N^{pz}^C)Audmpz **4**	572.3
(C^N^{py}^C)Audmpz	557.3
(C^N^{pz}^C)AuC≡CPh **5a**	535.3
(C^N^{py}^C)AuC≡CPh	505.4
[**H-5a**]$^+$	706.7

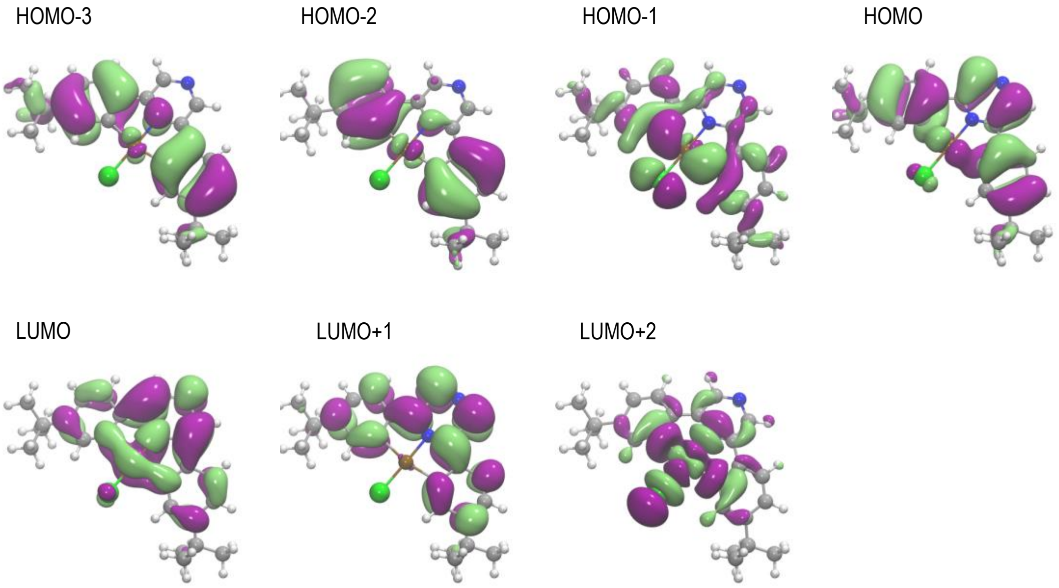

Figure S30. The frontier orbitals of complex **2**.

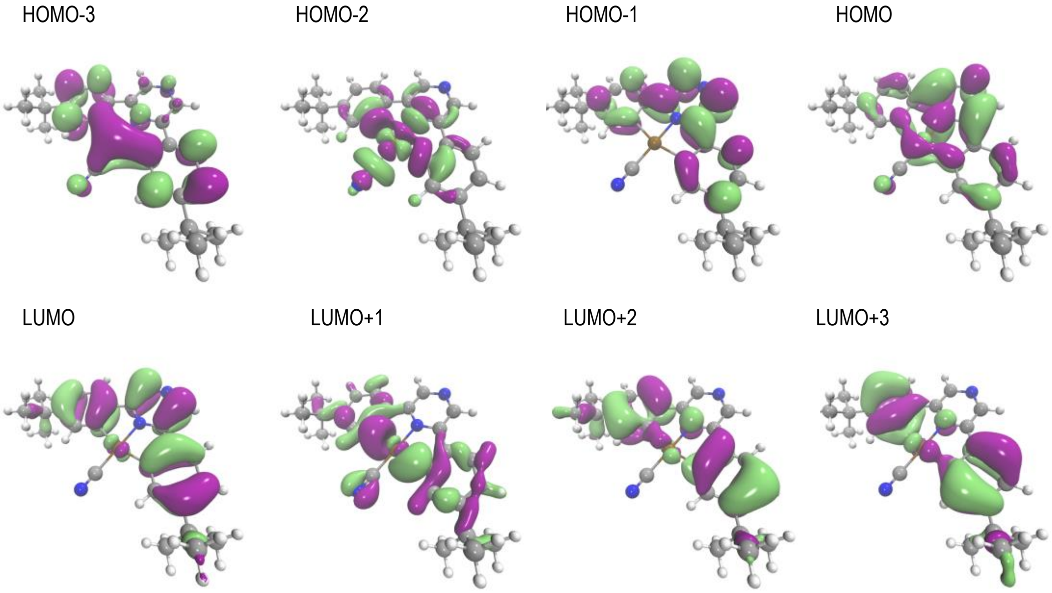

Figure S31. The frontier orbitals of complex **3**.

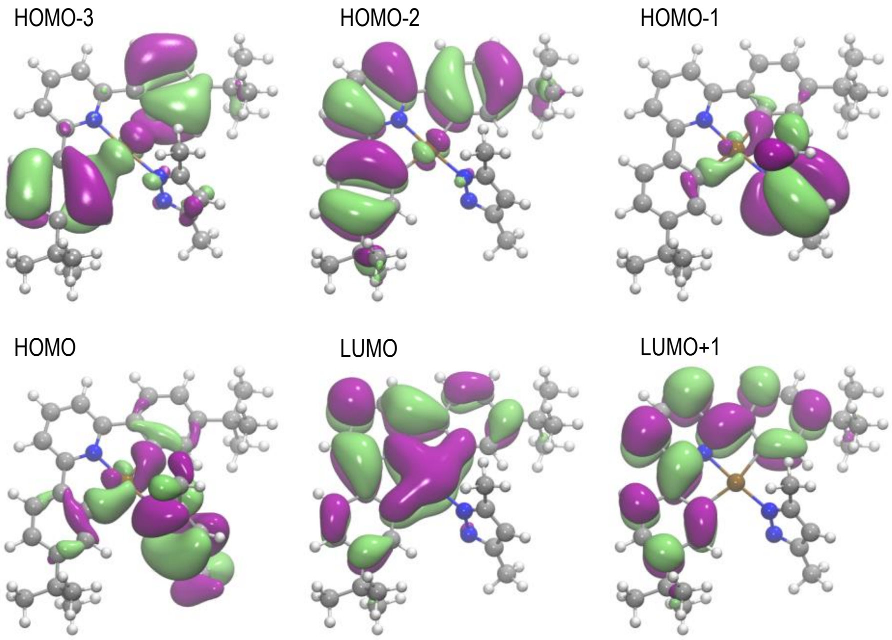

Figure S32. The frontier orbitals of complex **4**.

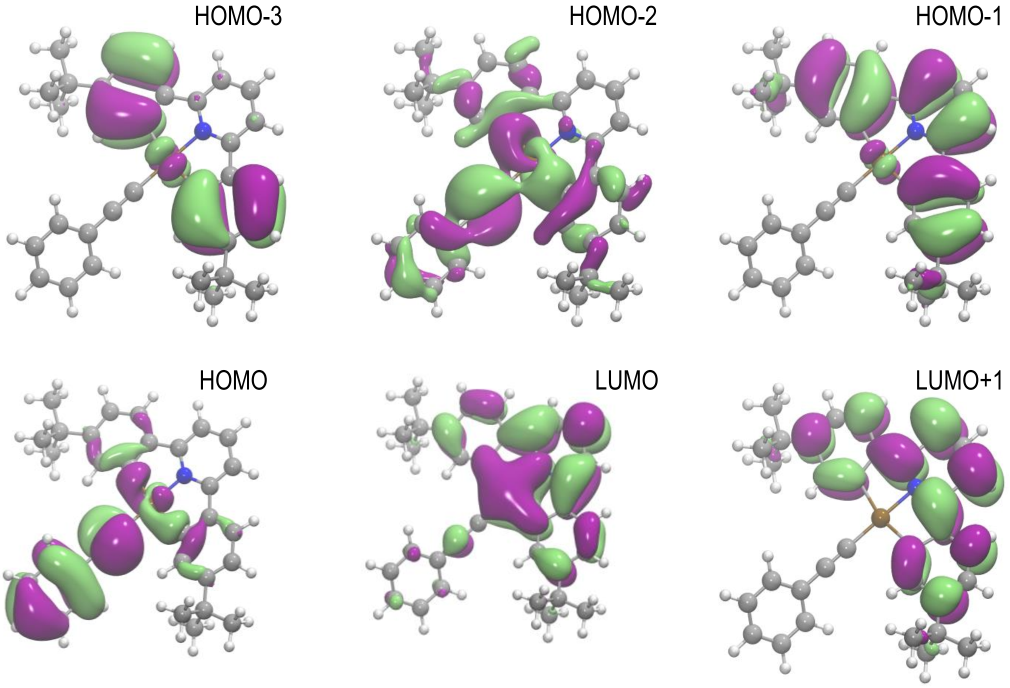

Figure S33. The frontier orbitals of complex **5a.**

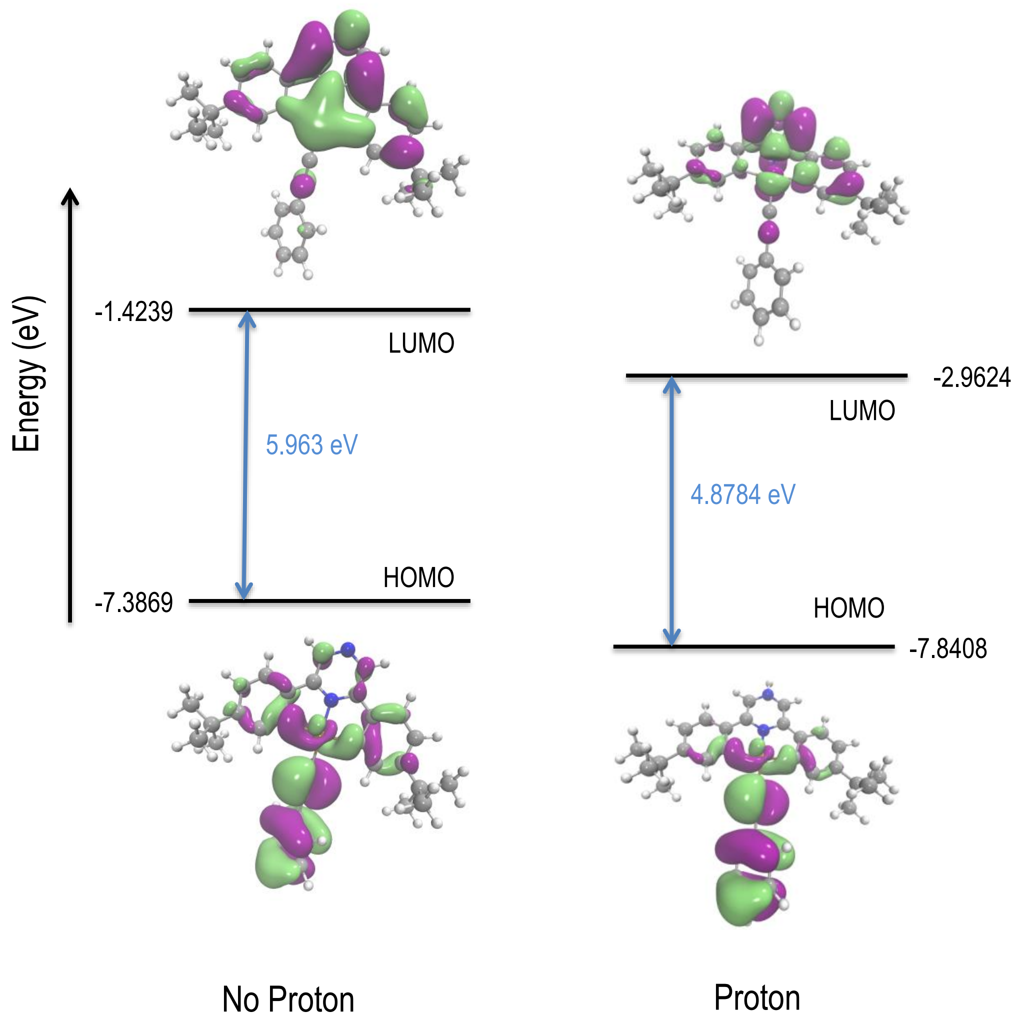

Figure S34. The effect of protonation on the frontier orbitals of **5a**.

S5. References

S1 N. Schultheiss, E. Bosch, *Heterocycles* 2003, **60**, 1891-1897.

S2 *Programs CrysAlisPro*, Oxford Diffraction Ltd., Abingdon, UK (2010)

S3 G. M. Sheldrick, *Acta Cryst.* 2008, **A64**, 112.

S4 A. L. Spek, (2006) PLATON – A Multipurpose Crystallographic Tool, Utrecht University, Utrecht, The Netherlands. A. L. Spek, *Acta Cryst.* 1990, **A46**, C34

S5 L. J. Farrugia, *J. Appl. Crystallogr.* 1999, **32**, 837.

S6 '*International Tables for X-ray Crystallography'*, Kluwer Academic Publishers, Dordrecht. Vol. C. 1992, pp. 500, 219 and 193.

S7 U. Resch-Genger, K. Rurack. *Pure Appl. Chem*. 2013, **85**, 2005-2026. A. M. Brouwer. *Pure Appl. Chem*. 2011, **83**, 2213-2228 and references therein.

S8 J. P. Perdew, K. Burke, and M. Ernzerhof, '*Generalized gradient approximation made simple*' *Phys. Rev. Lett*. 1996, **77**, 3865. C. Adamo and V. Barone, '*Toward reliable density functional methods without adjustable parameters: The PBE0 model*' *J. Chem. Phys*. 1999, **110**, 6158-69.

S9 F. Neese, '*The ORCA Program System*'. *WIREs Comput. Mol. Sci*. 2012, **2**, 73−78.

S10 F. Weigend and R. Ahlrichs, '*Balanced basis sets of split valence, triple zeta valence and quadruple zeta valence quality for H to Rn: Design and assessment of accuracy*' *Phys. Chem. Chem. Phys*. 2005, **7**, 3297 and F. Weigend, '*Accurate Coulomb-fitting basis sets for H to Rn*' *Phys. Chem. Chem. Phys*., 2006, **8**, 1057-65.

S11 C. Ullrich, '*Time-Dependent Density-Functional Theory*'; Oxford Graduate Texts: Oxford, U.K., 2011.

S12 S. Hirata, M. Head-Gordon, '*Time-Dependent Density Functional Theory Within the Tamm−Dancoff Approximation'*. *Chem. Phys. Lett*. 1999, **314**, 291−299.

S13 R. Baer, E. Livshits, U. Salzner, '*Tuned Range-Separated Hybrids in Density Functional Theory*'. *Ann. Rev. Phys. Chem*. 2010, **61**, 85−109.

S14 H. Iikura, T. Tsuneda, T. Yanai, K. Hirao, '*A Long-Range Correction Scheme for Generalized-Gradient-Approximation Ex- change Functionals*'. *J. Chem. Phys*. 2001, **115**, 3540−3544.

S15 O. S. Bokareva, G. Grell, S. I. Bokarev, O. Kühn, '*Tuning Range-Separated Density Functional Theory for Photocatalytic Water Splitting Systems'*. *J. Chem. Theory Comput*. 2015, **11**, 1700−1709.
